# Diverging repeatomes in holoparasitic Hydnoraceae uncover a playground of genome evolution

**DOI:** 10.1111/nph.70280

**Published:** 2025-06-14

**Authors:** Woorin Kim, Nicola Schmidt, Matthias Jost, Elijah Mbandi Mkala, Sylke Winkler, Guangwan Hu, Tony Heitkam, Stefan Wanke

**Affiliations:** ^1^ Department of Botany and Molecular Evolution Senckenberg Research Institute and Natural History Museum 60325 Frankfurt am Main Germany; ^2^ Institute of Ecology, Evolution and Diversity Goethe‐Universität Frankfurt 60438 Frankfurt am Main Germany; ^3^ Institute of Biology I RWTH Aachen University 52074 Aachen Germany; ^4^ CAS Key Laboratory of Plant Germplasm Enhancement and Specialty Agriculture, Wuhan Botanical Garden Chinese Academy of Sciences Wuhan 430074 China; ^5^ Sino‐Africa Joint Research Center Chinese Academy of Sciences Wuhan CN‐430074 China; ^6^ University of Chinese Academy of Sciences Beijing CN‐100049 China; ^7^ Dresden‐Concept Genome Center 01307 Dresden Germany; ^8^ Max Planck Institute of Molecular Cell Biology and Genetics 01307 Dresden Germany; ^9^ Departamento de Botánica Universidad Nacional Autónoma de México 04510 Mexico City Mexico

**Keywords:** comparative genomics, DNA transposons, parasitic plants, repetitive DNA, ribosomal DNA, transposable elements

## Abstract

- The transition from an autotrophic to a heterotrophic lifestyle is associated with numerous genomic changes. These often involve large genomic alterations, potentially driven by repetitive DNAs. Despite their recognized role in shaping plant genomes, the contribution of repetitive DNAs to parasitic plant genome evolution remains largely unexplored. This study presents the first analysis of repetitive DNAs in Hydnoraceae genomes, a plant family whose members are holoparasitic.
- Repetitive DNAs were identified and annotated *de novo*. Abundant transposable elements and 35S ribosomal DNA in the *Hydnora visseri* genome were reconstructed *in silico*. Their patterns of abundance and presence–absence were individually and comparatively analyzed.
- Both Hydnoraceae genera, *Hydnora* and *Prosopanche*, exhibit distinct repeatome profiles which challenge our current understanding of repeatome and rDNA evolution. The *Hydnora* genomes are dominated by long terminal repeat retrotransposons, while the *Prosopanche* genomes vary greatly in their repeat composition: *Prosopanche bonacinae* with a highly abundant single DNA transposon and *Prosopanche panguanensis* with over 15% 5S rDNA, compared to ≤ 0.1% in the *Hydnora* genomes.
- The repeat profiles align with the phylogeny, geographical distribution, and host shifts of the Hydnoraceae, indicating a potential role of repetitive DNAs in shaping Hydnoraceae genomes to adapt to the parasitic lifestyle.

## Introduction

Parasitic plants, representing *c*. 1% of angiosperms (Nickrent, 2020), have taken an evolutionary turn from autotrophy to heterotrophy, which entailed considerable genomic alterations in the nuclear as well as the organelle genomes. Holoparasitic plants, which completely lack photosynthetic activity, possess a highly reduced plastid genome (plastome) due to a widespread loss of genes involved in photosynthesis and housekeeping (Lyko & Wicke, 2021; Kim *et al*., 2023). Mitochondrial genomes of parasitic plants display diverse evolutionary patterns across parasitic lineages, such as extensive gene losses in mistletoe (*Viscum* spp.; Zervas *et al*., 2019), maintenance of the core mitochondrial gene set in dodders (*Cuscuta* spp.; Petersen *et al*., 2020), as well as high structural complexity alongside many integrations of foreign sequences in the Balanophoraceae (Zhou *et al*., 2023). Nuclear genomic studies are available for a limited number of parasitic lineages, suggesting deeply intertwined host–parasite relations (Davis & Wurdack, 2004; Yang *et al*., 2019), accelerated evolutionary rates (Lemaire *et al*., 2011; Bromham *et al*., 2013), and massive convergent gene loss with concomitant gene enrichment associated with the parasitic invasion (Sun *et al*., 2018; Xu *et al*., 2022; Ashapkin *et al*., 2023; Chen *et al*., 2023). Furthermore, genome size changes and chromosomal rearrangements (Piednoël *et al*., 2015; Neumann *et al*., 2021), genes associated with transposable element (TE)‐like domains (Cai *et al*., 2021), and horizontal TE transfer (Sun *et al*., 2018; Yoshida *et al*., 2019) suggest a potential role of repetitive DNAs in shaping these genomes.

Repetitive DNAs (repeats) are a major component of all plant nuclear genomes (Orozco‐Arias *et al*., 2019). Repeats significantly contribute to genome dynamics by driving mutations in coding sequences, influencing chromosomal organization, and affecting genome size (Raskina *et al*., 2008; Lee & Kim, 2014; Mehrotra & Goyal, 2014; Anderson *et al*., 2019; Schmidt *et al*., 2024). Studies on parasitic plant genomes uncovered long terminal repeat (LTR) retrotransposons of the Ty3‐*gypsy* and Ty1‐*copia* types as the most abundant repeats in the majority of studied lineages (Weiss‐Schneeweiss *et al*., 2006; Piednoël *et al*., 2012, 2015; Yoshida *et al*., 2019; Becher *et al*., 2021; Neumann *et al*., 2021), mirroring patterns seen in autotrophic plants (Orozco‐Arias *et al*., 2019; Wells & Feschotte, 2020). However, DNA transposons were found to be the most abundant repeats within certain parasitic plant genomes (Cai *et al*., 2021). LTR retrotransposons were identified as the major cause of genome variation in *Orobanche* and *Phelipanche* (Piednoël *et al*., 2012), whereas satellite DNAs influence the size of *Cuscuta* genomes (Neumann *et al*., 2021). In addition, the loss of particular Ty3‐*gypsy* LTR retrotransposons may be associated with the formation of holocentric chromosomes (Neumann *et al*., 2021). Nuclear ribosomal DNAs, important for both ribosome biogenesis and chromosome behavior as nucleus organization regions (Rogers & Bendich, 1987; Kubis *et al*., 1998; Kobayashi, 2011), show accelerated evolutionary rates in mycoheterotrophic, hemiparasitic, as well as holoparasitic angiosperm lineages (Nickrent & Starr, 1994; Lemaire *et al*., 2011; Bromham *et al*., 2013). Given the potential impact of repeats on genome dynamics, further investigation on the repetitive fraction of the genome (repeatome) within and across parasitic plant lineages is essential to investigate their role in parasitic plant genome evolution (Lyko & Wicke, 2021).

**Fig. 1:**
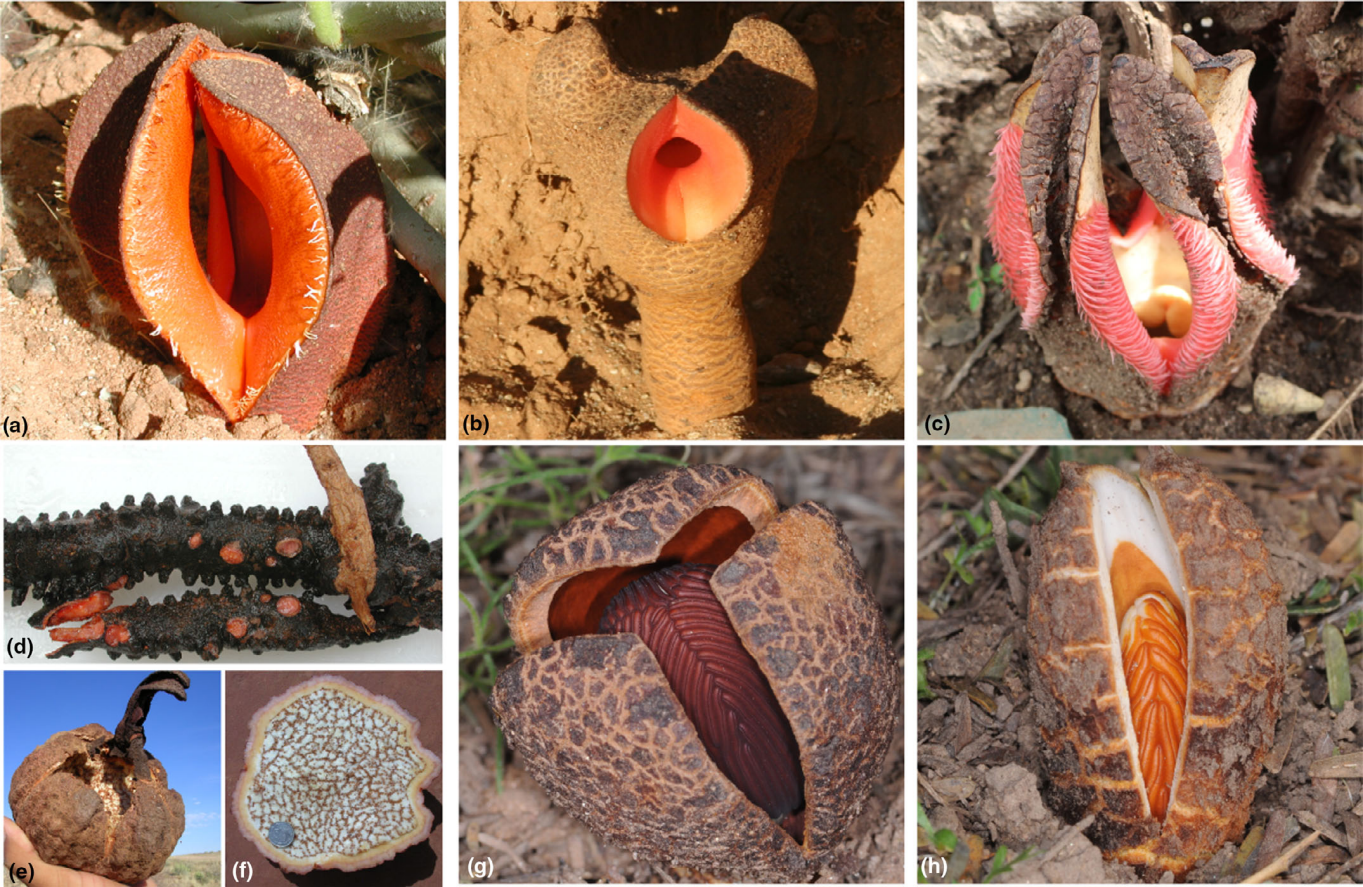
Flowers, fruit, and rhizome of Hydnoraceae; (a) *Hydnora visseri* flower; (b) *Hydnora triceps* flower; (c) *Hydnora abyssinica* flower; (d) *Hydnora visseri* rhizome; (e) *Hydnora visseri* fruit; (f) *Hydnora visseri* fruit section; (g) *Prosopanche americana* flower; (h) *Prosopanche bonacinae* flower. Photographs (a, d, e, f) by Jay Bolin, (b) by Stefan Wanke, (c) by Elijah Mbandi Mkala (g, h) by Andrea Cocucci. (g, h) were reproduced from Jost *et al*. (2022).

Following from the above work, our focus has been upon Hydnoraceae, a family of root holoparasites within the magnoliid order Piperales (Fig. 1; Nickrent *et al*., 2002; Jost *et al*., 2021), whose nuclear genomes still remain to be explored. Hydnoraceae comprise the two genera *Hydnora* (Thunberg, 1775) and *Prosopanche* (de Bary, 1868), with an estimated crown group age of *c*. 58 million years ago (Naumann *et al*., 2013) or older, tracing back to the continental separation as suggested by their distribution (Jost *et al*., 2022). *Hydnora* species are distributed from Southern Africa to the Arabian Peninsula (Decaisse, 1873; Musselman & Visser, 1989; Bolin & Musselman, 2013; Hatt *et al*., 2024b), while *Prosopanche* species are found in South and Central America (Cocucci, 1965; Gómez *et al*., 1981; Cocucci & Cocucci, 1996; Machado & De Queiroz, 2012; Martel *et al*., 2018; De Carvalho *et al*., 2021). Most common host plant species of Hydnoraceae are members of the Fabaceae and Euphorbiaceae; additionally, up to 16 other families have been reported as hosts (Hatt *et al*., 2023, 2024a). Recent taxonomic studies recognize 10 species of *Hydnora* and seven species of *Prosopanche*, with a more finely resolved classification dividing the genus *Hydnora* into four subgenera (Hatt *et al*., 2023, 2024b). Their unusual lifestyle as root holoparasites, for example, spending most of their lifetime underground (Tennakoon *et al*., 2007), and their extremely reduced morphology suggest distinctive evolutionary dynamics in their genomes.

Molecular genomic studies on Hydnoraceae are scarce, primarily due to the limited availability of plant material. None of the species are currently in cultivation, likely because of their strict dependence on host plants for growth and survival. Only a few cultivation attempts have been reported: one describing the germination of *Hydnora triceps* using host root extract (Bolin *et al*., 2009), and another documenting growth in a pot though without fruit production (Musselman & Visser, 1989) – possibly due to the absence of suitable pollinators (Bolin *et al*., 2009; Rocamundi *et al*., 2024). An additional attempt to cultivate *Hydnora africana* in a botanical garden was unsuccessful (Thorogood, 2019). The lack of cultivation limits access to fresh material for molecular studies, such as genome size measurements. Genome sizes of Hydnoraceae remain yet unknown although we attempted the estimation using *k*‐mer‐based approaches, flow cytometry, and comparative DAPI (4′,6‐diamidino‐2‐phenylindole) staining. Nonetheless, existing molecular genetic research suggests an accelerated evolutionary rate in the nuclear‐encoded small‐subunit rDNA (18S ribosomal DNA; Nickrent & Duff, 1996; Lemaire *et al*., 2011; Bromham *et al*., 2013). The plastid genomes of Hydnoraceae are highly reduced, and phylogenetic analysis of their sequences results in extremely long branches, even when compared to other parasitic plants (Naumann *et al*., 2013; Jost *et al*., 2020). On the other hand, the draft mitochondrial genome of *Hydnora visseri* is four‐fold larger than those of related species (Yu *et al*., 2023).

Here, we aim to illuminate the genome evolution of holoparasitic Hydnoraceae, focusing on the repeats. We hypothesize that the dispersed and tandemly repeated DNAs (Kubis *et al*., 1998), including TEs, satellite DNAs, and ribosomal DNAs, facilitate genome dynamics in Hydnoraceae genomes (Oliver & Greene, 2009) and may contribute to the adaptation to the holoparasitic lifestyle. To study this, we analyze low coverage Illumina short reads of eight *Hydnora* and three *Prosopanche* species, which cover *c*. 80% and 40% of the species diversity within the respective genus. We used read clustering to deduce the individual repeat profiles of the 11 Hydnoraceae, using *H. visseri* as a reference species. Additional long reads of *H. visseri* allowed us to reconstruct representative sequences of the most abundant repetitive DNAs and to provide detailed repeat classifications, serving as a basis for a comparative repeatome analysis of the family. Furthermore, the ribosomal DNAs were analyzed in‐depth to elucidate the sequence variation and genomic organization, as well as the variable genomic abundance within and between both *Hydnoraceae* genera.

## Materials and Methods

### Plant material, DNA extraction and genome sequencing

In this work, we re‐examined whole genome sequencing data from the following studies: Naumann *et al*. (2013); Jost *et al*. (2020, 2022); and Mkala *et al*. (2023). For the information regarding the DNA extraction and short read sequencing details, see Supporting Information Table S1. For the long reads of *Hydnora visseri* Bolin, E. Maass & Musselman, DNAs were extracted using the CTAB method (Doyle & Doyle, 1987), modified to add 4 μl of RNase A (10 mg ml^−1^; Thermo Fisher Scientific, Waltham, MA, USA) from silica‐dried material as described in Naumann *et al*. (2016). One continuous long‐read single molecule real‐time (SMRT) Pacific Biosciences library was prepared from high‐molecular‐weight genomic DNA, following the ‘20 kb template preparation using Blue Pippin™ size selection system’ protocol (version P/N 100‐286‐000‐07). The final large‐insert library was selected for fragments larger than 10 kb using the BluePippin™ device (Sage Science Inc., Beverly, MA, US). This PacBio library was sequenced on 43 PacBio RS2 SMRT cells v3 for 6 h using DNA polymerase P6 and sequencing chemistry 4.0.

### Global repeat analysis by read clustering

To prepare the input data for read clustering analyses, the quality of each set of Illumina reads was first evaluated using FastQc v.0.11.5 (Andrews, 2010). Subsequent read pre‐processing involved several steps, including filtering, trimming, and random subsampling: Plastome sequences and Illumina adapters were filtered using Bowtie2 v.2.2.6 (Langmead & Salzberg, 2012). From each forward‐ and reverse‐read dataset, five million reads were randomly sampled and trimmed to 100 nucleotides (nt) using Trimmomatic v.0.39 (Bolger *et al*., 2014). Finally, the reads were merged into a paired‐end read file per species, labeled with species‐specific codes (see Table S1). For a comparative purposes, a close photoautotrophic relative of the Hydnoraceae, *Aristolochia fimbriata* Cham. (Aristolochiaceae, SRR13748080) underwent the same procedure and was included in the analysis. For the visual overview of the processes, see Fig. S1A.

Read clustering analyses were performed using the RepeatExplorer2 pipeline (RE2; Novák *et al*., 2013) applying default parameters (90% similarity over 55% of the read length, cluster size threshold = 0.01% of the analyzed reads, minimal cluster size for assembly = 5). The REXdb (Viridiplantae v.3.0; Neumann *et al*., 2019) database served as a reference for identifying TE protein domains for primary annotations. In addition, a comparative analysis was performed using a concatenated dataset of 1 million randomly subsampled reads for each species from the datasets used for individual read clustering analysis. A custom repeat database containing the most abundant repeats of the *H. visseri* genome (see next paragraph) was included for the individual and comparative read clustering analyses.

### *In silico* reconstruction of repeat references

Seventeenth reference repeats were reconstructed for the most abundant read clusters, exceeding 0.2% of the analyzed reads, in the *H. visseri* genome (Table S2). To achieve this, the highest read‐depth contigs of the corresponding read clusters provided by the pipeline were used for a basic local alignment search (BLASTn embedded in Geneious v.6.1.8; Kearse *et al*., 2012) against *H. visseri* PacBio long reads (Table S2). The searched 20 best‐matching long reads, representing the top 20 BLASTn hits with the lowest e‐values, were investigated using self‐ and pairwise dotplots based on the presence of conserved TE protein domains or ribosomal RNA genes. The 20 full or fragmented repeats were then reconstructed as a consensus using Muscle alignments (Edgar, 2004), providing a reference repeat. Lastly, the conserved protein domains harbored by the reference repeats were annotated using Domain‐based ANnotation of Transposable Elements (DANTE) (Novák *et al*., 2024). These reconstructed repeats were collected to create a custom repeat database for the annotation of repeats during subsequent RE2 analyses. For a visual overview of the processes, see Fig. S1B.

Further species‐specific repetitive elements were reconstructed or obtained, including an unclassified repeat found in the genomes of *H. hanningtonii* Rendle, *H. abyssinica* A. Braun, and *H. solmsiana* Dinter. The highest read‐depth contigs from the most abundant unclassified read cluster of each species (provided by the RE2 pipeline) were refined by mapping all available short reads of the respective species to each contig using Bowtie2, generating consensuses. These three consensuses were aligned using Muscle (for alignment, see Fig. S2). Two unclassified repeats in the *H. esculenta* genome were reconstructed using the same method. In addition, a *P. panguanensis* C. Martel & Rob. Fern.‐specific satellite DNA and two *P. bonacinae* Speg.‐specific satellite DNA candidates were obtained as consensus sequences provided by the RE2 pipeline (for alignment, see Fig. S3). The reconstructed repeats as well as the consensuses provided by the RE2 pipeline can be accessed in a public data repository (see the ‘Data availability’ section).

### *In silico* reconstruction of Hydnoraceae 5S ribosomal RNA genes

For only three of the investigated Hydnoraceae species (*H. triceps* Drège & E. Mey., *P. bonacinea*, and *P. panguanensis*), consensuses for the 5S rDNA were provided by the pipeline. These RE2 consensuses were refined by mapping the short reads from the aforementioned species against the respective RE2 consensus using Bowtie2 v.2.2.6 (Langmead & Salzberg, 2012). The refined 5S rDNAs were used for the reconstruction of the 5S rDNA of the remaining Hydnoraceae species: Short reads of the *Hydnora* species were mapped individually against the 5S rDNA reference from *H. triceps*, whereas *P. americana* short reads were mapped against the 5S rDNA reference from *P. panguanensis* using Bowtie2 v.2.2.6. The respective number and proportion of mapped reads are listed in Table S3. The secondary structures of the reconstructed Hydnoraceae 5S rRNA genes were predicted using the RNAstructure software (Reuter & Mathews, 2010; Fig. S4). In addition, *H. visseri* transcriptomes from six individual tissues (osmophore, tepal, stamen, fruit, rhizome, and gynoecium, N. Pabón‐Mora, F. González, C. W. de Pamphilis, J. F. Bolin, C. Neinhuis, J. F. Alzate, & S. Wanke, unpublished) were mapped to the reconstructed *H. visseri* 5S rDNA, using Bowtie2 v.2.2.6 (Langmead & Salzberg, 2012).

To compare the reconstructed Hydnoraceae 5S rDNAs with those from 56 other angiosperms, we extracted the individual 5S rDNA consensus from available genome assemblies or whole genome sequencing data (Table S4). For each angiosperm species, a 5S rDNA consensus sequence was generated from the alignment of 50 5S rDNA sequences extracted using Blast, using the *A. fimbriata* 5S rDNA gene as a query sequence. This query sequence was reconstructed using *A. fimbriata* Nanopore long reads (accession no. SRR13748081), based on a Mafft alignment (Katoh *et al*., 2002) of 50 5S DNA sequences identified from the *A. fimbriata* long reads. For some species, 5S rDNAs were retrieved from a publicly available rRNA database (Szymanski *et al*., 2002; http://combio.pl/rrna/), instead. The sequence similarity of all 5S rDNAs to each other was visualized as a heatmap generated using R v.4.4.0. (R Core Team, 2021), with a distance matrix based on the Kimura‐2‐parameter model (Kimura, 1980) using the R packages ape (Paradis & Schliep, 2019) and pheatmap (Kolde, 2018). In addition, the relative rates among the 5S rDNA of *H. visseri* and closely related species of the Piperales were estimated using Mega11 (Table S5; Tamura *et al*., 2021) based on Tajima's method (Tajima, 1993).

Additionally, a complete 5S monomer (including the 5S rRNA gene and the non‐transcribed spacer (NTS)) was reconstructed for *H. visseri*. To do so, *H. visseri*‐derived PacBio long reads were mapped to the *H. visseri* 5S rDNA (see previous section) using Bowtie2 v.2.2.6 (Langmead & Salzberg, 2012). Repetitive 5S monomers were identified on 37 long reads by inspecting self‐ and pairwise dotplots. In total, 248 partial and full‐length monomers were extracted and aligned using Muscle to create the consensus sequence of the complete 5S rDNA monomer. The arrangement of the 5S rDNA monomers on the PacBio long reads was investigated using Blast (Altschul *et al*., 1990; threshold E‐value 1E‐5). The Blast hits on each long read were visualized with a zoom factor of 500 on each self‐dotplot created by FlexiDot (word size 10; Seibt *et al*., 2018).

## Results

### The *H. visseri* genome is dominated by Ty3‐*gypsy* retrotransposons of the Tekay and Ogre type

**Fig. 2:**
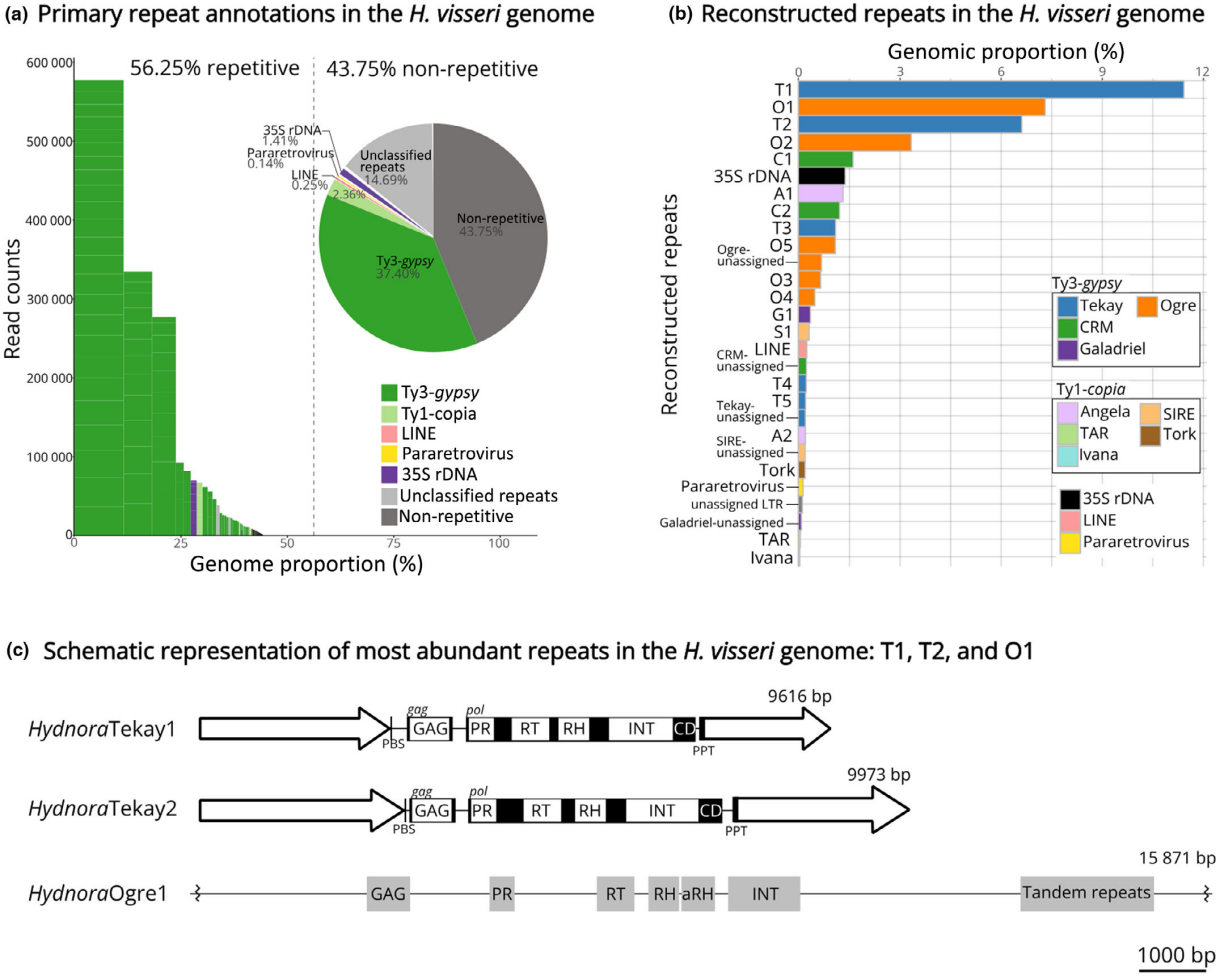
Reconstruction of the most abundant repetitive elements in the *Hydnora visseri* genome. (a) The relative proportion of repetitive elements in the *H. visseri* genome, primarily annotated by the RepeatExplorer2 pipeline. Bars indicate superclusters, with each stack within the bar representing a read cluster. The height and width of the bars correspond to the read counts of the supercluster and the genomic proportion, respectively. The proportions are calculated based on the number of reads in each category divided by the total number of analyzed reads. (b) Genomic proportions of reconstructed repeats from the *H. visseri* genome. The bar length represents the genomic proportions, calculated as the percentage of reads within the corresponding clusters divided by the total number of analyzed reads. Abbreviations of the individual LTR retrotransposons are as follows: *Hydnora*Tekay1‐5 (T1–T5), *Hydnora*Ogre1‐5 (O1–O5), *Hydnora*CRM1‐2 (C1–C2), *Hydnora*Angela1‐2 (A1–A2), *Hydnora*Galadriel1 (G1), *Hydnora*SIRE1 (S1; Supporting Information Table S2). Bars labeled as ‘unassigned’ and further categories (i.e. ‘Tork’, ‘Pararetrovirus’, ‘TAR’, and ‘Ivana’) correspond to the totality of elements belonging to the respective category and falling below the abundance threshold for the reconstruction of reference elements (proportions were summed). (c) Schematic representation of most abundant repeats in the *H. visseri* genome. Long terminal repeats (LTRs) are represented by arrows at the 5′ and 3′ ends of the elements. The protein domains were annotated using Domain‐based ANnotation of Transposable Elements (DANTE), using default setting (REXdb: Viridiplantae_v.3.0; Scoring Matrix: BLOSUM80). The shaded black blocks behind the protein annotations of *Hydnora*Tekay1 and *Hydnora*Tekay2 refer to the open reading frames (ORFs), where the *gag* and *pol* ORFs are separated. The zigzag lines on the 5′ and 3′ ends of *Hydnora*Ogre1 indicate truncated ends. aRH, archeal ribonuclease H; CD, chromodomain; GAG, gag‐like protein; INT, integrase; PBS, primer binding site; PPT, polypurine tract; PR, protease; RH, ribonuclease H; RT, reverse transcriptase.

To gain insights into the genomic repeat profiles of *Hydnoraceae*, low‐coverage genomic short reads of *H. visseri* were clustered initially to retrieve the most abundant repetitive DNAs. This ultimately provided a repeat database containing the most abundant repetitive elements from the *H. visseri* genome (Fig. 2). Using this database, Hydnoraceae repeat profiles were annotated, allowing for an in‐depth individual and comparative genomic analysis. For our reference taxon *H. visseri*, it is estimated that over half of its genome consists of repetitive sequences (56.3%; Fig. 2a). Ty3‐*gypsy* retrotransposons, particularly Tekay and Ogre elements, are the most abundant repeats, accounting for two‐thirds of the *H. visseri* repeatome (Fig. 2a,b). Reference sequences were reconstructed for the 17 most abundant repeats, including *Hydnora*Tekay1‐4 (T1, T2, T3, T4), *Hydnora*Ogre1‐5 (O1, O2, O3, O4, O5), *Hydnora*CRM1 and 2 (C1, C2), *Hydnora*Angela1 and 2 (A1, A2), *Hydnora*SIRE1 and 2 (S1, S2), *Hydnora*Galadriel1 (G1), and a complete 35S rDNA monomer comprising the 18S, 5.8S, 26S genes alongside intergenic spacers (Table S2). Among these 17 repeats, 12 belong to the Ty3‐*gypsy* retrotransposons (i.e. Tekay, Ogre, CRM, and Galadriel). A few Tekay and Ogre elements predominate the repeatome (T1, O1, T2; Fig. 2b), with the reconstructed sequences of the two Tekay elements retaining *gag* and *pol* open reading frames (Fig. 2c). On the other hand, Ty1‐*copia* retrotransposons account for 2.4% of the *H. visseri* genome, represented mainly by Angela and SIRE (Fig. 2b; Table S2). The 35S rDNA contributes 1.4% to the genome, whereas 5S rDNA was not detected by the RE2 pipeline. These detailed annotations of *H. visseri* read clustering analysis using the reconstructed repeats provided a basis to further annotate other Hydnoraceae repeatomes.

### Repeatomes of further *Hydnora* species mirror those of *H. visseri*, whereas *Prosopanche* repeatomes are highly divergent

**Fig. 3:**
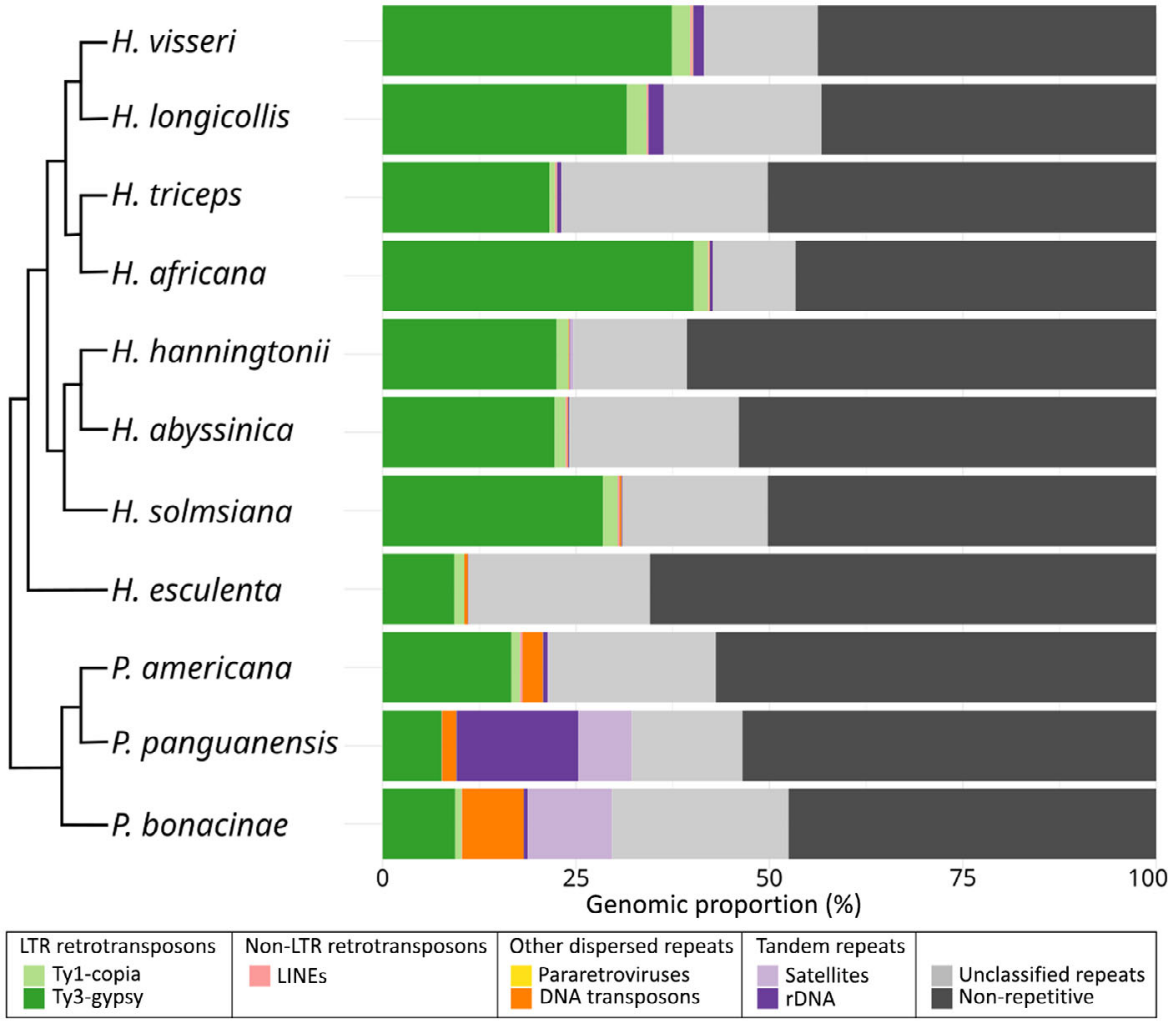
Repeat profiles of the analyzed Hydnoraceae genomes: Genomic abundance of repetitive elements from the individual read clustering analysis using the RE2 pipeline (Supporting Information Tables S5, S6). One bar indicates the entirety of one genome, where the stacks indicate the respective repeat composition. The cladogram is adapted from Mkala *et al*. (2023), modified. *Hydnora visseri*, *Hydnora longicollis*, *Hydnora triceps*, *Hydnora africana*, *Hydnora hanningtonii*, *Hydnora abyssinica*, *Hydnora solmsiana*, *Hydnora esculenta*, *Prosopanche americana*, *Prosopanche panguanensis*, *Prosopanche bonacinae*.

**Fig. 4:**
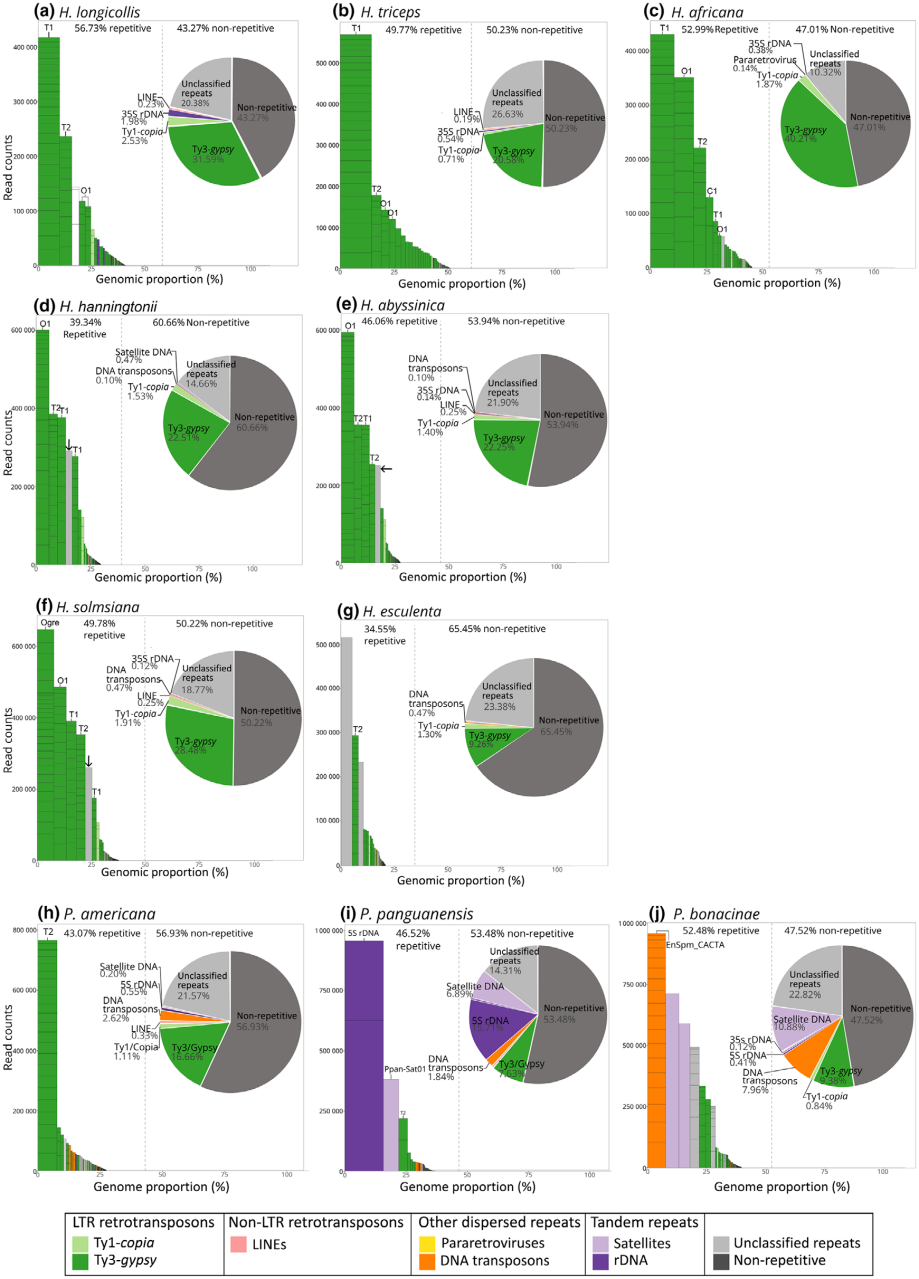
Repeat profiles of seven *Hydnora* and three *Prosopanche* genomes (a–g): Repeat profiles of *Hydnora* genomes excluding the aforementioned *Hydnora visseri* genome; (h–j) Repeat profiles of *Prosopanche* genomes. Bars indicate the superclusters, with each stack representing a read cluster. The height and width of the bars correspond to the read counts within the supercluster and the genomic proportion, respectively. Superclusters were annotated using the reference repeat database of 17 reconstructed elements from the *H. visseri* genome. For *Hydnora longicollis* (a), the third bar in the bar chart (colored in white) refers to a contamination of the input read dataset by short nucleotide sequences containing G‐ and A‐stretches. *Hydnora longicollis*, *Hydnora triceps*, *Hydnora africana*, *Hydnora hanningtonii*, *Hydnora abyssinica*, *Hydnora solmsiana*, *Hydnora esculenta*, *Prosopanche americana*, *Prosopanche panguanensis*, *Prosopanche bonacinae*.

To capture the genome dynamics represented in repeat compositions, the individual repeatomes of the *Hydnora* and *Prosopanche* genomes were analyzed and annotated using the repeat database from the *H. visseri* read clustering analysis (Figs 3, 4). The *Hydnora* genomes show variability in their overall repeat content, ranging from 34.6% in *H. esculenta* to 56.7% in *H. longicollis* (Fig. 3; Table S2). Ty3‐*gypsy* retrotransposons are the most abundant repeats in all analyzed *Hydnora* genomes (9.3–40.2%; Table S6). In particular, different Ty3‐*gypsy* elements predominate in the genomes: Tekay elements are most abundant in the genomes of *H. visseri* (T1), *H. longicollis* (T1), *H. triceps* (T1), *H. africana* (T1), and *H. esculenta* (T2), while Ogre elements predominate in the genomes of *H. hanningtonii* (O1), *H. abyssinica* (O1), and *H. solmsiana* (Ogre; Fig. 4a–g). The highly abundant unclassified repeats in the genomes of *H. hanningtonii*, *H. abyssinica*, and *H. solmsiana* turned out to be the same repeat present in all three genomes (indicated as arrows in Fig. 4d–f; for the alignment, see Fig. S2). The *H. esculenta* genome contains two additional, highly abundant unclassified repeats (Fig. 4g). Aside from the high abundance of Ty3‐*gypsy* retrotransposons, *Hydnora* genomes are characterized by scarce amounts of 5S rDNA, DNA transposons, and satellite DNAs (Fig. 4a–g; Table S6). By contrast, these elements are much more abundant in the *Prosopanche* genomes (Fig. 4h–j). 5S ribosomal DNA is the most prevalent repeat in the *P. panguanensis* genome, followed by a satellite DNA (Ppan_Sat01; Fig. 4i; Table S7). The En/Spm_CACTA superfamily of DNA transposons, which contains a DDE‐type transposase (Fig. S3), is the most abundant repeat in the *P. bonacinae* genome, followed by two similar satellite DNA candidates and Ty3‐*gypsy* retrotransposons (Figs 4j, S5). Ty3‐*gypsy* retrotransposons, particularly the T2 element, predominate in the *P. americana* genome (Fig. 4h), and also contribute to the remaining *Prosopanche* genomes.

### Relative repeat abundances mirror the Hydnoraceae phylogeny

**Fig. 5:**
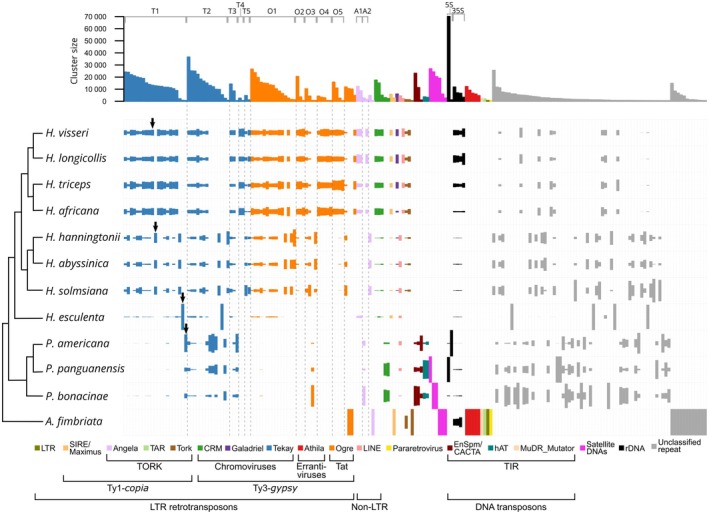
Comparative genomic repeat composition among 11 *Hydnoraceae* species and *Aristolochia fimbriata*. The size of each rectangle is proportional to the genomic abundance of the repeat in the respective species. Dashed vertical lines group read clusters that belong to the same repeat, while black arrows indicate taxa‐specific sequence variants of *Hydnora*Tekay1. In the top bar chart, the height of each bar represents the number of reads in each cluster comprising at least 516 reads (≥ 0.01% of the analyzed reads). Clusters corresponding to the same repetitive element are sorted by read count, from highest to lowest, so the bar chart depicts the ranked abundance of each repetitive element. The cladogram is adapted from Mkala *et al*. (2023). For the modified version of this figure that includes cluster identifiers; see Supporting Information Fig. S6. *Hydnora visseri*, *Hydnora longicollis*, *Hydnora triceps*, *Hydnora africana*, *Hydnora hanningtonii*, *Hydnora abyssinica*, *Hydnora solmsiana*, *Hydnora esculenta*, *Prosopanche americana*, *Prosopanche panguanensis*, *Prosopanche bonacinae*.

**Fig. 6:**
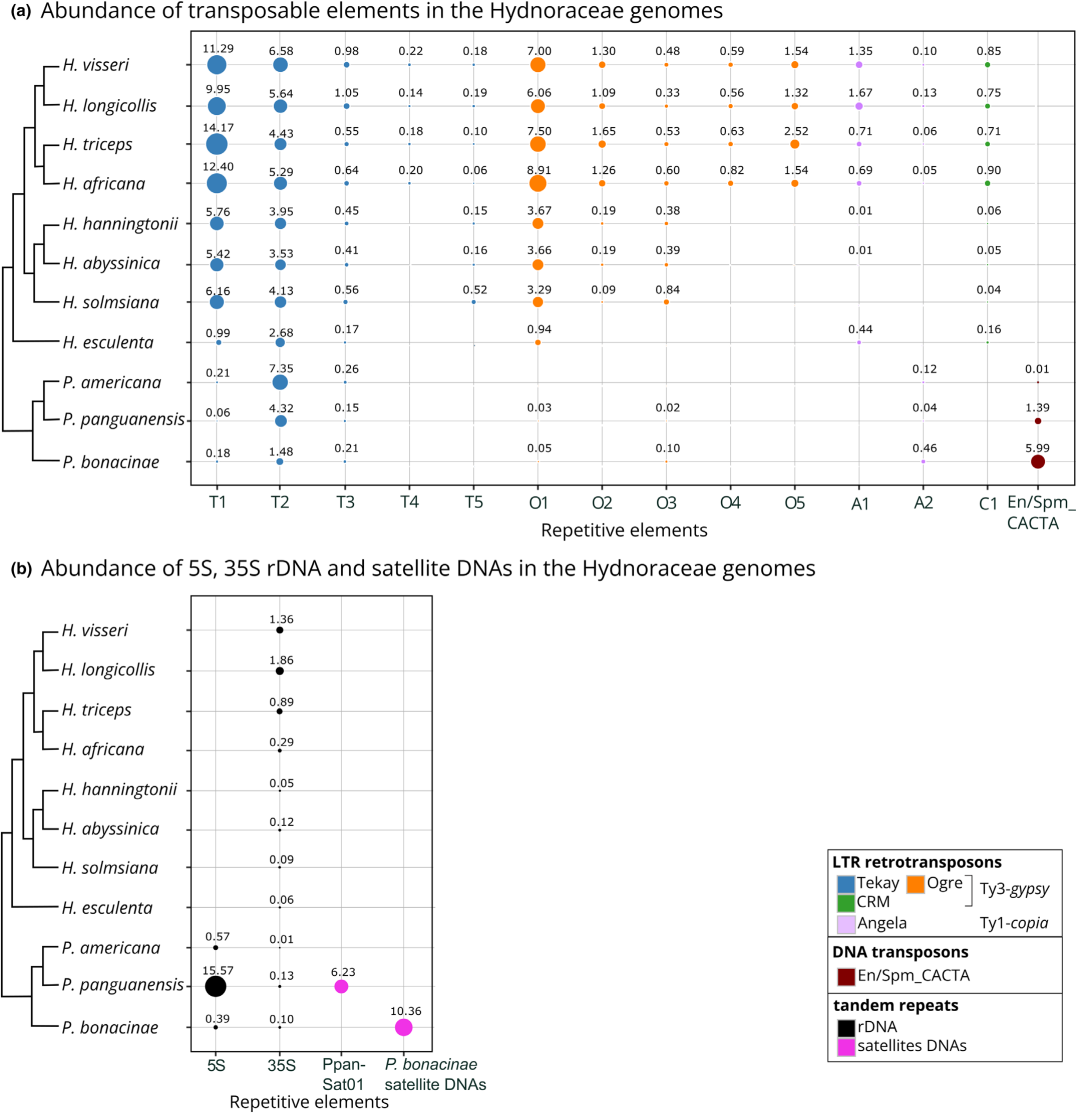
Comparative abundance of the repetitive elements in the Hydnoraceae genomes. (a) The abundance of reconstructed TEs. (b) The abundance of 5S, 35S rDNA, and satellite DNA. The size of each bubble indicates the relative abundance of the repeats among the analyzed species. This is calculated as the read count of a repetitive DNA present in a genome divided by the total number of reads clustered for that repeat from the comparative read clustering analysis (see Supporting Information Table S8 for the proportions). The numbers above the bubble indicate the repeat abundance within the respective genome, calculated as the same read counts divided by the total number of analyzed reads for that species, thus representing the genome proportion as a percentage (%). The cladogram is adapted from Mkala *et al*. (2023).

To provide a comparative overview of the repeat profiles of Hydnoraceae, subsets of reads from all studied Hydnoraceae species were pooled for a joint read clustering analysis. The read clusters were then annotated using the *H. visseri* repeats database, which allowed for the identification of repeats shared across the analyzed Hydnoraceae genomes. Overall, the repeat profiles reflect the phylogenetic relationships: Phylogenetically close *Hydnora* species, that is species in the same clade, share the patterns of read cluster abundance, identified by protein domain similarity (Fig. 5). Taxon‐specific repeats were observed as well; for instance, T4, O4, and O5 are exclusively detected in the genomes of *H. visseri*, *H. longicollis*, *H. triceps*, and *H. africana* (Fig. 6; Table S8). *Hydnora esculenta*, sister to the remaining *Hydnora* species, exhibits unique sequence variations within otherwise shared retrotransposons (e.g. T1 and T2; Fig. 5). Such variations, reflected in the presence or absence of certain read clusters (indicated by black arrows in Fig. 5), are observed across all reconstructed repeats. *Prosopanche* repeat profiles vary to a higher degree compared to those of its sister genus *Hydnora*, particularly, but not only when examining the distribution of unclassified repeats (shown in gray in Fig. 5). The presence of DNA transposons, 5S rDNA, and species‐specific satellite DNAs characterizes the *Prosopanche* genomes (Fig. 6; Table S8). Noticeably, the 5S rDNA clusters are composed almost entirely of reads from the *P. panguanensis* genome (Table S8), in contrast to the 35S ribosomal DNA, which are relatively evenly distributed and more abundant in some *Hydnora* species (Fig. 6; Table S8). Finally, *A. fimbriata*, the photoautotrophic species closely related to Hydnoraceae, displays a distinct repeat profile, where Athila and Ogre‐type retrotransposons (Ty3‐*gypsy*) are the most abundant TEs, with unique satellite DNAs, and many unclassified repeats (Fig. 5; Table S8). Summarizing, these diverging repeat profiles mirror the taxa's phylogeny and provide a solid basis for deeper investigation of specific repeat types—such as the 5S rDNA with its striking abundance differences.

### Hydnoraceae 5S rDNA sequences vary greatly from those of other angiosperms

**Fig. 7:**
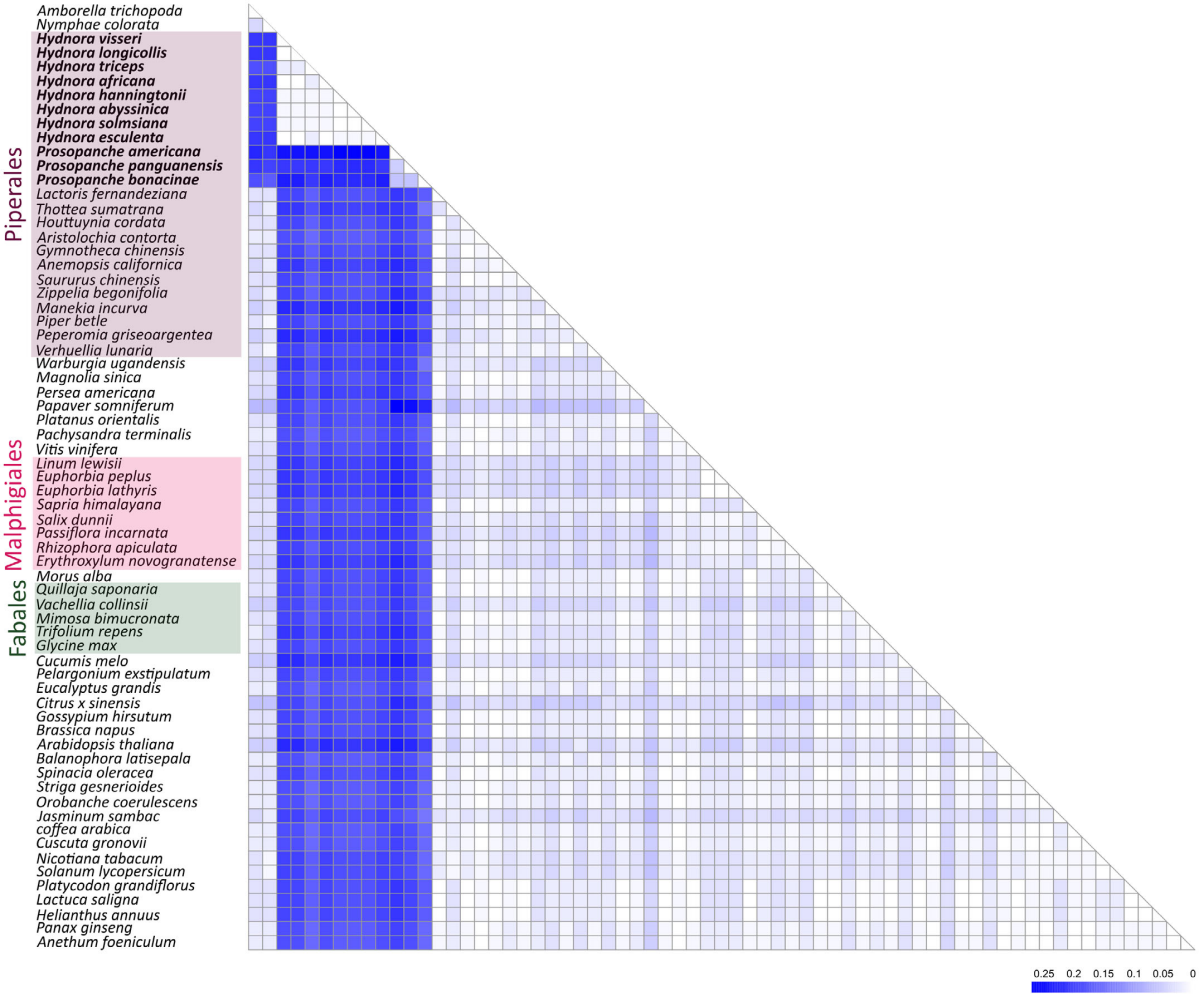
Genetic distances between the coding regions of Hydnoraceae 5S ribosomal DNAs and those of 56 other angiosperms, calculated using the Kimura‐2‐parameter model (Kimura, 1980). The resulting distance matrix ranges from 0 to 0.27, with higher values indicating greater sequence variation. Representatives of the order Piperales are highlighted in purple. Fabales and Malpighiales, the host orders, are highlighted in green and pink, respectively. Parasitic plants are highlighted in bold. For the modified figure to include genetic distance values, see Supporting Information Fig. S8.

Among all analyzed *Hydnora* genomes, we detected the 5S rDNA only in the *H. triceps* genome, and in very low quantities (Table S3). This observation could result from the 5S rDNA copies being generally low in abundance in the *Hydnora* genomes, potentially falling below the detection threshold of the RE2 pipeline, or due to a high sequence divergence in 5S rDNAs that impedes detection. In order to understand the low amount of 5S rDNA detected in *Hydnora* genomes, we reconstructed 5S rDNAs from all analyzed Hydnoraceae species using the 5S rDNA from the *H. triceps* genome. In comparison to an autotrophic relative, *Aritolochia fimbriata*, the total reads of *Hydnora* species mapped to the *H. triceps* 5S rDNA were quite low (0.00005%–0.003% vs 0.073%; Table S3). The reconstructed 5S rDNAs were then compared to those of other angiosperms (including additional Piperales, and the major host orders Malpighiales and Fabales) using a genetic distance matrix (Fig. 7). Genetic distances were calculated based on the 5S rDNAs sequence alignment across Hydnoraceae and 56 other angiosperms (Fig. 7, for alignment, see Fig. S7). With genetic distances greater than 0.15, indicating that over 15% of nucleotides were substituted, Hydnoraceae 5S rDNA sequences are largely divergent from those of other angiosperms (Figs 7, S8). Specifically, genetic distances between Hydnoraceae 5S rDNA and closely related families within the Piperales, including Lactoridaceae, Aristolochiaceae, Saururaceae, and Piperaceae, exceed 0.16. Furthermore, 5S rDNA from the major host lineages Malpighiales and Fabales show no particular similarity to Hydnoraceae 5S rDNAs, with genetic distances exceeding 0.19. Within Hydnoraceae, the genetic distance between *Hydnora* and *Prosopanche* 5S rDNAs ranges from 0.21 to 0.27, with 0.27 representing the highest genetic distance observed across all analyzed species.

These variations occur throughout the DNA sequence, but are less pronounced within the internal control region for transcription, specifically the A‐box, intermediate element, and C‐box (Fig. S7). In addition, despite such sequence variation, predicted secondary structures of Hydnoraceae 5S rRNA present the general helices I–IV and loops A–E of eukaryotic 5S rRNA, albeit with somewhat lower base‐pair probability compared to closely related autotrophic species (Fig. S4). The GC content of *Hydnora* 5S rRNAs ranges from 52.9 to 54.2%, similar to that of Aristolochiaceae (*Aristolochia*) 5S rRNAs (Fig. S4), while *Prosopanche* 5S rRNAs contain less G and C (42–45.5%). Overall, Hydnoraceae 5S rDNA sequences are highly divergent from those of other angiosperms and between the two genera, while secondary structures are relatively conserved.

### The 5S rDNA arrays in the *H. visseri* genome show a high degree of diversification

**Fig. 8:**
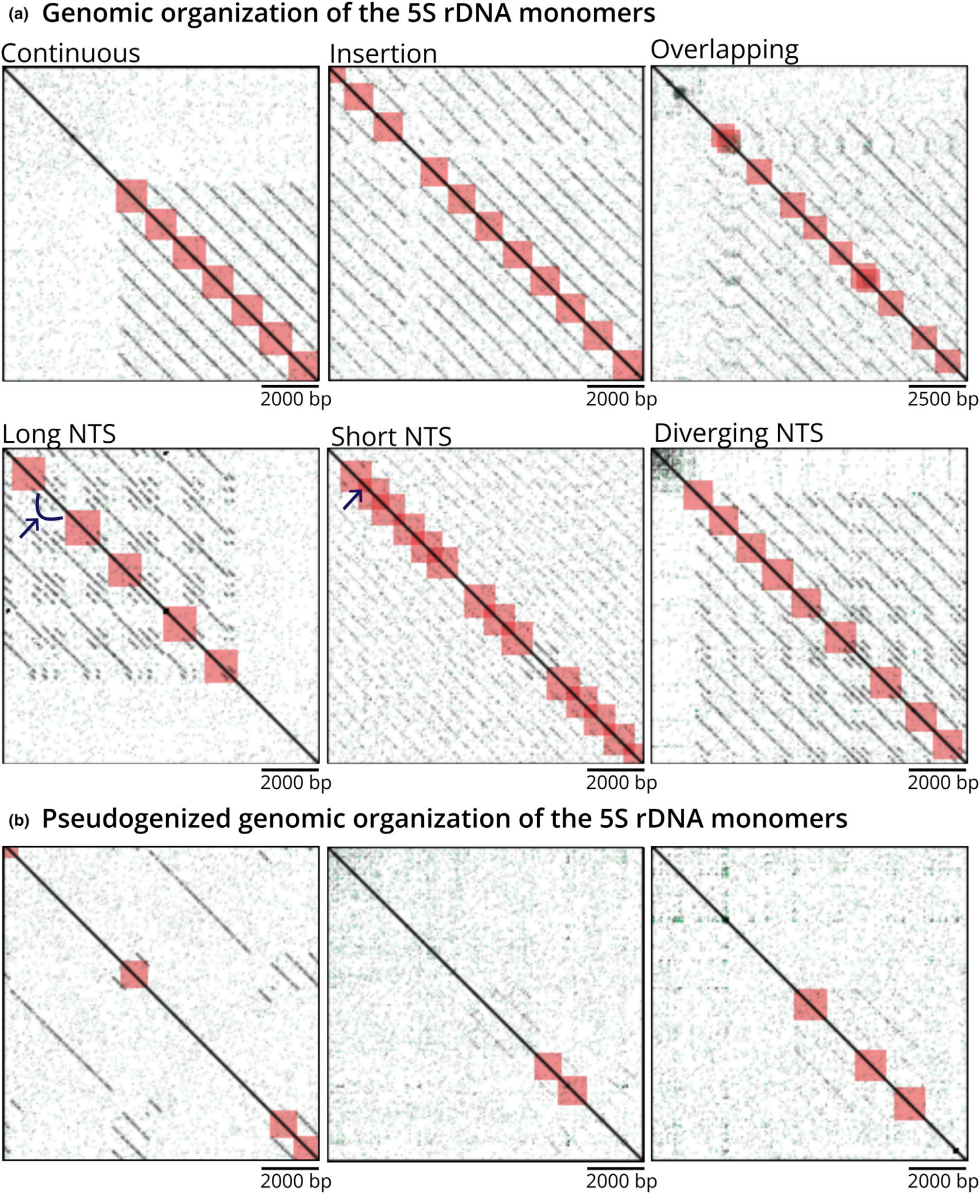
Genomic organization of *Hydnora visseri* 5S rDNA monomers. Self‐dotplots of PacBio long reads containing 5S rDNA arrays and monomers identified by BLAST (*E*‐value = 0.0001) are displayed. Black lines parallel to the main diagonal represent sequence repetitions. Red boxes indicate a BLAST hit corresponding to the 5S rDNA gene (121 bp), including 500 bp up‐ and downstream of the gene (annotation zoom = 500). Thus, the tandem repetitions of the monomers include the non‐transcribed spacers (NTS; 977 bp; a) indicating regular arrangements of monomers, including length variants of NTSs. The elongated and shortened NTSs are highlighted by arrows. (b) Displays potentially pseudogenized 5S rDNA arrangements (faint, non‐continuous lines in the dotplot), often exhibiting large gaps between monomers.

To capture the 5S rDNA organization within the *H. visseri* genome, a complete 5S rDNA monomer (121 bp gene and 977 bp NTS) was reconstructed and aligned to *H. visseri* long reads (Fig. 8). Among the 4540 941 long reads analyzed (*c*. 500–72 000 bp in length), 319 (0.007%) exhibit one or more 5S rDNA monomer(s). These monomers vary greatly in their organization. Regular 5S rDNA arrays, consisting of at least five monomers arranged without major gaps in the reads longer than 6000 bp, were identified 56 times (Fig. 8a). Some regular arrays exhibit sequence divergence or monomer rearrangements, such as insertions or overlapping monomers (Fig. 8a). Moreover, length‐variants of the NTSs were observed, recognizable by differing distances between the individual 5S rDNA monomers (Fig. 8a, highlighted by arrows). The elongated NTSs are due to internal sequence repetitions, as displayed in the dotplots (Fig. 8a; Long NTS). Additionally, 24 potentially regular arrays were identified; however, due to the short read length (< 6000 bp), their integrity could not be confirmed. A total of 239 instances were identified as pseudogenic, characterized by large gaps between monomers due to sequences falling below the detection threshold (*E*‐value = 0.0001; Fig. 8b). These pseudogenized arrays also showed high sequence variation, as indicated by faint lines within the dotplots.

## Discussion

In this study, we present the first global repeatome analysis of Hydnoraceae and examine its evolutionary implications. Despite limitations such as the lack of genome size estimates, which may lead to underestimation of repeat content due to technical thresholds in the clustering analysis, we identify notable patterns in the repeatomes. We highlight the potential evolutionary roles of abundant LTR retrotransposons, which are commonly observed in other parasitic plant genomes, as well as the rather unusual dominance of 5S rDNA and DNA transposons in certain *Prosopanche* species. We provide a broader context to the highly variable and scarce *Hydnora* 5S rDNAs through comparison with rDNAs in other parasitic plants and by reviewing 5S rDNA studies across angiosperms. Finally, we discuss the repeatome landscape with regard to phylogeny, geographical distribution, and host plant shifts, exploring the potential role of repetitive DNA in genomic adaptation.

### The diversified Hydnoraceae repeat landscape suggests a potential role of repeats in genome dynamics

As in many studied seed plants (Wessler *et al*., 1995; Wells & Feschotte, 2020), including parasitic plants (Piednoël *et al*., 2012; Yoshida *et al*., 2019; Becher *et al*., 2021; Neumann *et al*., 2021), LTR retrotransposons are the main contributors to the *Hydnora* repeatomes. Ty3‐*gypsy* TEs represent the most abundant LTR retrotransposon superfamily across all *Hydnora* and *Prosopanche americana* genomes, while they rank second in abundance in the other two *Prosopanche* species analyzed. On average, Ty3‐*gypsy* retrotransposons are 15.7 times more prevalent in the *Hydnora* genomes than those belonging to Ty1‐*copia*, which is remarkable even taking into account that angiosperms generally contain more Ty3‐*gypsy* (17–30%) compared to Ty1‐*copia* retrotransposons (6–10%; Giraud *et al*., 2021). This large Ty3‐*gypsy* fraction is dominated by a few high copy repeats, in particular two Tekay‐ and one Ogre‐type repeats, suggesting their rapid accumulation in the recent past (Feschotte *et al*., 2002). Theses abundant Tekay and Ogre repeats are worth investigating further in relation to their impact on genome size and chromosome configuration, as (1) abundant TEs often correlate positively with genome size (Leeton & Smyth, 1993; Lee & Kim, 2014; Jayakodi *et al*., 2023; Ishiguro *et al*., 2025), also observed in parasitic plants (Cai *et al*., 2021; Neumann *et al*., 2021); and as (2) Ty3‐*gypsy* elements often play major roles in chromosomal behaviour and heterochromatin silencing (Bourque *et al*., 2018; Orozco‐Arias *et al*., 2019).

Two of the *Prosopanche* genomes exhibit significantly different repeat profiles compared to those of *Hydnora* and *P. americana*. They harbor not only fewer LTR retrotransposons, but also highly abundant satellite DNAs compared to *Hydnora* genomes (Tables S6, S7). In addition, high 5S rDNA abundance in *P. panguanensis* and the enrichment of En/Spm_CACTA (a DNA transposon) in *P. bonacinae* deserves further attention. 5S rDNA, as the most abundant repetitive element in a genome, is to our knowledge unprecedented and could impede chromosomal stability via frequent recombination (Raskina *et al*., 2008; Ding *et al*., 2022). Similarly, DNA transposons are generally less abundant in plant genomes as compared to LTR retrotransposons (Feschotte & Pritham, 2007), due to silencing by the host genome, cut‐and‐paste transposition (vs retrotransposons' copy‐and‐paste), and retrotransposon interference targeting their catalytic region (Zhao *et al*., 2016; Liu *et al*., 2021). Nevertheless, active En/Spm_CACTA elements could transpose into the proximity of the coding regions of genes and can play a major role in genome divergence as observed in rice, cabbage, and maize (Jiang *et al*., 2003; Alix *et al*., 2008; Yang *et al*., 2013). Notably, DNA transposon abundance has been observed in a holoparasite before: in the endoparasite *Sapria himalayana* (Rafflesiaceae), two abundant DNA transposon families dominate the repeatome, which comprise 90% of the genome assembly, and a high number of predicted genes contain TE‐like domains (Cai *et al*., 2021). In addition, rapid evolution through actively amplifying DNA transposons has been observed occasionally across the tree of life, for example, within genomes of taxa with rapid speciation records such as bats (Mitra *et al*., 2013; Paulat *et al*., 2022). Thus, the abundant En/Spm_CACTA DNA transposon in the *P. bonacinae* genome is worth investigating further in terms of a potential repeat‐driven evolution.

Summarizing, the Hydnoraceae repeat profiles are highly divergent, suggesting potential repeat‐driven genomic alteration. This could involve locus‐specific gene mutations via transposition of particular repeats into coding regions or broader genomic changes such as genome size fluctuation and chromosome rearrangements (Piednoël *et al*., 2012; Neumann *et al*., 2021). Given the extensive gene loss and gene family reduction observed in parasitic plants – particularly in the root holoparasite *Balanophora* (Chen *et al*., 2023) and endoparasite *Sapria* (Cai *et al*., 2021) – such repeat‐driven dynamics may have played a role in Hydnoraceae genome evolution. Further validation is required, such as refined assessment of repeat presence and abundance in *Hydnora* and *Prosopanche* genomes following the estimation of genome size, and using for example fluorescent *in situ* hybridization to validate and visualize specific repetitive elements at the chromosomal level.

### Rapidly evolving Hydnoraceae 5S rDNA and its scarcity in *Hydnora* genomes challenge our current understanding

Ribosomal RNA genes are typically under strong selective pressure due to their importance in translation. However, recent studies suggest that ribosomal DNA sequence heterogeneity may be more common in eukaryotes than previously thought (Wang *et al*., 2023). In angiosperms, 5S rDNA variants are observed at different chromosomal loci, originated from hybridization (Cloix *et al*., 2000; Fulnecek *et al*., 2002; Simon *et al*., 2018; Vozárová *et al*., 2021; Tynkevich *et al*., 2022; Chen *et al*., 2024). In Hydnoraceae genomes, rapidly evolving 5S rDNAs and high nucleotide substitutions raise questions about how well rRNA secondary structure, and thus function, is preserved. Despite the large number of nucleotide substitutions, the secondary structures are predicted to be generally intact, owing to complementary mutations (Fig. S4). This represents selective pressure on ribosomal DNA to preserve the secondary structure (Dixon & Hillis, 1993), exhibiting compensatory mutations or isosteric ‘wobble’ pairs (Stombaugh *et al*., 2009). Such a pattern was also observed in structure maintenance of rapidly evolving *Rafflesia keithii* (Rafflesiaceae) 18S rRNA (Nickrent & Starr, 1994). In order to further verify 5S rRNA functionality, transcriptomes of six different tissues from *H. visseri* were searched for 5S rRNA transcripts. A small number of transcripts (17–95 per tissue) were identified and aligned with the 5S rDNA reference. While the alignments did not fully recover conserved regions, the mere presence of 5S rRNA transcripts may hint toward the functionality. Still, Hydnoraceae 5S rDNAs leave open questions, for example the potential causes for sequence divergence, which may lie on polymerase fidelity (Loeb & Kunkel, 1982) and DNA replication and repair efficiency (Topal & Fresco, 1976) as suggested by Nickrent & Starr (1994).

One of the most striking features of the *Hydnora* 5S rDNA is its low genomic abundance. Although the actual 5S rDNA copy numbers remain to be explored, the low abundance contrasts with the typically larger 5S rDNA copy numbers observed in autotrophic plants, that is, a few hundred to more than 100 000 per haploid genome (Danna *et al*., 1996; Fulneček *et al*., 2002; Wang *et al*., 2019). In addition, recent studies reported that the gene families related to DNA repair and ribosome assembly are expanded rather than contracted in the holoparasitic *Balanophora* and the hemiparasitic *Striga asiatica* genomes (Yoshida *et al*., 2019; Chen *et al*., 2023), despite the general pattern of regressive evolution (Sun *et al*., 2018; Yoshida *et al*., 2019; Cai *et al*., 2021; Xu *et al*., 2022; Chen *et al*., 2023). Thus, whether the low genomic abundance of 5S rDNA in *Hydnora* genomes is lineage‐specific or represents a broader trend in parasitic plant genomes remains an open question. However, the lowest 5S copy number per genome is described in duckweed (46 5S rDNA copies; Chen *et al*., 2024), with its genome known to be rather ancient and small (158 Mb; Cao *et al*., 2016) and the plant having a reduced morphology. In addition, due to their organization in tandem, rDNAs are thought to evolve concertedly (Cronn *et al*., 1996; Garcia *et al*., 2024), with recombination as one of the driving forces (Rogers & Bendich, 1987; Raskina *et al*., 2008; Kobayashi, 2011). In *Hydnora* genomes, we not only observed a low overall abundance of rDNA but also identified multiple organizational variants of the 5S rDNA in *H. visseri*. These include highly irregular patterns of organization, which may represent remnants of once more extensive and cohesive arrays, characterized by sporadic and discontinuous monomer repetitions. It is conceivable, though it remains to be determined, whether the recombinogenic nature of the 5S rDNA contributes to such large‐scale alterations in its genomic organization.

Several key questions remain to be explored: As the genome size of the investigated plants is currently unknown, what would be the absolute 5S rDNA amount in *Hydnora* genomes? If *Hydnora* genomes were as big as assumed (> 10 GB, dePamphilis, personal communication), would low 5S rDNA abundance be sufficient for ribosome biogenesis, or may it affect cell growth and proliferation (Moss, 2004; Qi *et al*., 2016; Shore & Albert, 2022)? Alternatively, are *Hydnora* ribosomes actually composed of 5S rRNA with an alternate sequence, possibly benefiting from the rRNA or other ribosomal components of their host (Yang *et al*., 2019) or are they replaced by an alternative structure, as observed in the mammalian and yeast mitochondrial ribosomes (Amunts *et al*., 2014; Brown *et al*., 2014; Greber *et al*., 2014)? We argue that investigating the transcriptome and proteome, especially in terms of ribosome architecture, will promote understanding of how Hydnoraceae holoparasites execute the basic genetic processes of transcription and translation. At the same time, studies of further holo‐ or hemiparasitic plants will provide an opportunity to place the findings of this study into a broader evolutionary context.

### Hydnoraceae repeat profiles reflect their phylogeny, geographic distribution, and host shifts

The repeat landscape across the analyzed Hydnoraceae species is largely congruent with a recent plastid‐based Hydnoraceae phylogeny (Mkala *et al*., 2023), mirrored by patterns of shared repeat abundance in closely related species as well as the presence of (sub‐)genus‐specific repeats. The species of the subgenus *Hydnora* (represented here by *H. visseri*, *H. longicollis*, *H. triceps*, and *H. africana*) (Decaisse, 1873; Hatt *et al*., 2024b) share the same Tekay element as their most abundant repeat (T1) and harbor several clade‐specific elements (T4, O4, and O5; Fig. 6). These species are exclusively found in southern Africa (mostly South Africa, Namibia, and southern Angola) and parasitize *Euphorbia* spp. (Decaisse, 1873; Musselman & Visser, 1989; Hatt *et al*., 2024b). By contrast, the species of the subgenus *Dorhyna*, represented here by *H. hanningtonii*, *H. abyssinica*, and *H. solmsiana*, are distributed in eastern and northern Africa, up to the Arabian peninsula, and parasitize *Fabaceae* spp. (Decaisse, 1873; Musselman & Visser, 1989; Hatt *et al*., 2024b). Compared to the subgenus *Hydnora*, these species exhibit Ogre retrotransposons as the most abundant repeats, along with further clade‐specific repeat (Fig. S2). They also show sequence divergence in the shared repeats, represented by the patterns of read cluster distribution (Fig. 5). *Hydnora esculenta*, endemic to Madagascar and the sole member of the subgenus *Neohydnora* (Harms, 1935; Bolin & Musselman, 2013), exhibits a unique repeat profile that is lacking most of the Tekay and Ogre retrotransposons found in other *Hydnora* species and harbors neither DNA transposons nor satellite DNAs. This results in the overall lowest repeat content observed among the studied species (34.55%; Table S6). While the other *Hydnora* species inhabit arid and semi‐arid climates, *H. esculenta* is also found in transitional rainforest areas (Bolin & Musselman, 2013; Hatt *et al*., 2024b). The distinct habitat may be correlated with genomic changes reflected in the repeat profile, for example, genetic drift, reduced gene flow due to population isolation, and stress‐induced TE activation (Casacuberta & González, 2013). Considering host shifts among genera, emerging questions include the possible impact of horizontal repeat transfers from host to parasite (Baidouri *et al*., 2014), and how these might have shaped *Hydnora* genomes.

In contrast to *Hydnora*, the geographic distribution of *Prosopanche* species does not closely align with their phylogeny. For instance, although *P. americana* and *P. panguanensis* are sister species, they do not occur in sympatry. Whereas, *P. panguanensis* inhabits the tropical rainforest of Peru (Martel *et al*., 2018), *P. americana* and *P. bonacinae* are both found in the arid regions of Argentina. Given that only 40% of known *Prosopanche* species were analyzed in this study, compared to 80% of the known *Hydnora* species diversity, the limited sampling may restrict a more comprehensive analysis, particularly as new species are likely to be discovered (Hatt *et al*., 2023). In addition, considering that most Hydnoraceae species are found in semi‐arid climates, the habitat of *P. panguanensis* in rainforests is again noticeable with regard to its repeatome: The unique, massive abundance of 5S rDNA may have contributed to the adaptation capacity of the species, since rDNA is known to represent recombinogenic ‘hot spots’, and could lead to speciation by chromosomal rearrangement, when combined with geographical isolation and establishment by genetic drift (Raskina *et al*., 2008; Rosato *et al*., 2015).

Taken together, this study contributes to the understanding of the nuclear genome in ‘the strangest plants in the world’, Hydnoraceae (Musselman & Visser, 1989), particularly with respect to repeatomes and their roles in genome evolution. The genomes of Hydnoraceae exhibit diverging repeat profiles, with abundant Tekay and Ogre (Ty3‐*gypsy*) LTR retrotransposons potentially affecting genomic organization. Abundant 5S rDNA and DNA transposons in each *Prosopanche* species are analyzed in terms of repeat‐driven evolution, whereas in *Hydnora*, the rapidly evolving, yet scarcely present 5S rDNA remains to be explored. In addition, we propose a possible link between geographical distribution, host shifts, and repeat compositions of Hydnoraceae, speculating that the accumulation patterns of repetitive elements may even contribute to genomic adaptation. We also want to pinpoint the limitations of our study, such as the scarcity of materials due to absence of cultivation, the lack of fresh material for genome size measurements, and hence a potential underestimation of repeats due to thresholds in the repeat clustering procedure. Nevertheless, these gaps underscore the significance of the present study, adding valuable insights into the evolution of parasitic plant nuclear genomes in general.

## Competing interests

None declared.

## Author contributions

S Wanke, TH conceived the study; data generation WK, NS, EMM, GWH, MJ, S Wanke, S Winkler; analyses WK, NS; writing of the first draft WK, NS; responsible for visualization of results WK, NS; all authors reviewed and edited the draft and agreed to the published version of the manuscript.

## Disclaimer

The New Phytologist Foundation remains neutral with regard to jurisdictional claims in maps and in any institutional affiliations.

## Supporting information


**Fig. S1** Workflow for the processing of the read data and the reconstruction of a preliminary reference database of transposable elements within the *Hydnora visseri* genome.
**Fig. S2** Alignment of the consensuses of the highly abundant unclassified read cluster from the *Hydnora abyssinica*, *Hydnora hanningtonii*, and *Hydnora solmsiana* individual analysis results.
**Fig. S3** Alignment of the transposase amino acid sequences of the highly abundant En/Spm_CACTA DNA transposon from the *Prosopanche bonacinae* genome and 16 further En/Spm_CACTA DNA transposon sequences.
**Fig. S4** Secondary structure of Hydnoraceae 5S rRNAs.
**Fig. S5** Alignment of the RE2‐provided consensuses from two highly abundant read clusters representing *Prosopanche bonacinae*‐specific satellite DNAs.
**Fig. S6** Comparative genomic repeat composition among 11 *Hydnoraceae* spp. and *Aristolochia fimbriata*, including the cluster IDs.
**Fig. S7** Alignment of 5S rDNAs of Hydnoraceae and of 56 further angiosperms 5S rDNAs.
**Fig. S8** Genetic distances between 5S rDNAs of Hydnoraceae and of 56 further angiosperms.
**Table S1** Plant material, DNA extraction and genome sequencing.
**Table S2** Reconstruction of repetitive elements within the *Hydnora visseri* genome.
**Table S3** Hydnoraceae short read mapping to 5S rDNA references.

**Table S4** NCBI identifiers for the genome sequencing data for the reconstructed angiosperm 5S rDNAs.
**Table S5** Relative rate test among 5S rDNA of *Hydnora visseri* and closely related species.
**Table S6** Summary of genomic proportions of repetitive elements in *Hydnora* genomes.
**Table S7** Summary of genomic proportions of repetitive elements in *Prosopanche* genomes.
**Table S8** Relative genomic abundance of specific Hydnoraceae repeats.
Please note: Wiley is not responsible for the content or functionality of any Supporting Information supplied by the authors. Any queries (other than missing material) should be directed to the *New Phytologist* Central Office.

## Data Availability

The dataset generated and/or analyzed during the current study is available in NCBI GenBank under the identifier: PRJNA1188088 (BioProject) and in the Zenodo repository doi: 10.5281/zenodo.14194251.
