# Supplementary material for: Diverging repeatomes in holoparasitic Hydnoraceae uncover a playground of genome evolution
Source: New Phytol. 2025 Jun 14;247(3):1520–37. doi: 10.1111/nph.70280 (PMC12222929; doi:10.1111/nph.70280)
Supplement: Supplementary file 1 — Fig. S1 Workflow for the processing of the read data and the reconstruction of a preliminary reference database of transposable elements within the Hydnora visseri genome. Fig. S2 Alignment of the consensuses of the highly abundant unclassified read cluster from the Hydnora abyssinica, Hydnora hanningtonii, and Hydnora solmsiana individual analysis results. Fig. S3 Alignment of the transposase amino acid sequences of the highly abundant En/Spm_CACTA DNA transposon from the Prosopanche bonacinae genome and 16 further En/Spm_CACTA DNA transposon sequences. Fig. S4 Secondary structure of Hydnoraceae 5S rRNAs. Fig. S5 Alignment of the RE2‐provided consensuses from two highly abundant read clusters representing Prosopanche bonacinae‐specific satellite DNAs. Fig. S6 Comparative genomic repeat composition among 11 Hydnoraceae spp. and Aristolochia fimbriata, including the cluster IDs. Fig. S7 Alignment of 5S rDNAs of Hydnoraceae and of 56 further angiosperms 5S rDNAs. Fig. S8 Genetic distances between 5S rDNAs of Hydnoraceae and of 56 further angiosperms. Table S1 Plant material, DNA extraction and genome sequencing. Table S2 Reconstruction of repetitive elements within the Hydnora visseri genome. Table S3 Hydnoraceae short read mapping to 5S rDNA references. [file NPH-247-1520-s002.pdf]

New Phytologist

***New Phytologist* Supporting Information**

Article title: Diverging repeatomes in holoparasitic Hydnoraceae uncover a playground of genome evolution

Authors: Woorin Kim, Nicola Schmidt, Matthias Jost, Elijah Mbandi Mkala, Sylke Winkler, Guangwan Hu, Tony Heitkam, Stefan Wanke

Article acceptance date: 18 May 2025

The following Supporting Information is available for this article:

Fig. S1 Workflow for the processing of the read data (A) and the reconstruction of a preliminary reference database of transposable elements within the *H. visseri* genome (B)

Fig. S2 Alignment of the consensuses of the highly abundant unclassified read clusters from the *H. abyssinica*, *H. hanningtonii*, and *H. solmsiana* individual analysis results

Fig. S3 Alignment of the transposase amino acid sequences of the highly abundant En/Spm_CACTA DNA transposon from the *P. bonacinae* genome and 16 further En/Spm_CACTA DNA transposon sequences

Fig. S4 Secondary structure of Hydnoraceae 5S rRNAs

Fig. S5 Alignment of the RE2-provided consensuses from two highly abundant read clusters representing *P. bonacinae*-specific satellite DNAs

Fig. S6 Comparative genomic repeat composition among eleven Hydnoraceae spp. and *A. fimbriata*, including the cluster IDs

Fig. S7 Alignment of 5S rDNAs of Hydnoraceae and of 56 further angiosperms

Fig. S8 Genetic distances between 5S rDNAs of Hydnoraceae and of 56 further angiosperms

Table S1 Plant material, DNA extraction and genome sequencing

Table S2 Reconstruction of repetitive elements within the *H. visseri* genome

Table S3 Hydnoraceae short read mapping to 5S rDNA references

Table S4 NCBI identifiers for the genome sequencing data for the reconstructed angiosperm 5S rDNAs (separate Excel file is provided)

Table S5 Relative rate test among 5S rDNA of *H. visseri* and closely related species

Table S6 Summary of genomic proportions of repetitive elements in *Hydnora* genomes

Table S7 Summary of genomic proportions of repetitive elements in *Prosopanche* genomes

Table S8 Relative genomic abundance of specific Hydnoraceae repeats

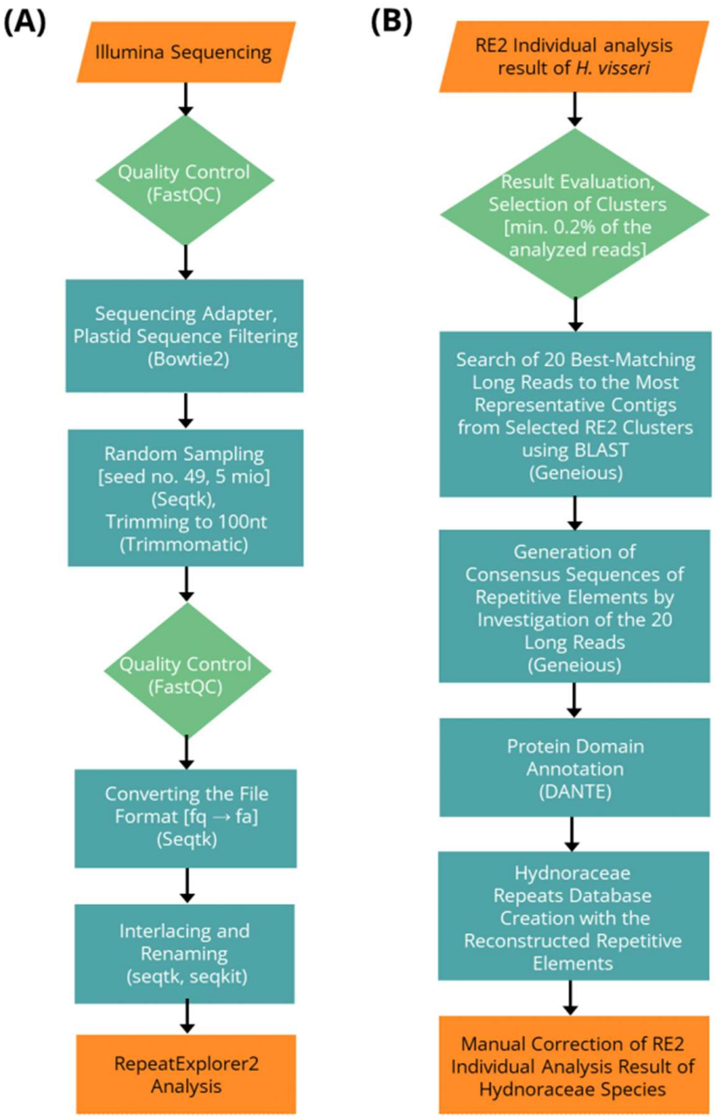

Fig. S1 Workflow for the processing of the read data (A) and the reconstruction of a preliminary reference database of transposable elements within the *H. visseri* genome (B). Parallelograms indicate input data, whereas rectangles and diamonds indicate processes and preparation steps, respectively. Light green rectangles indicate processes in which interim results were generated. (A) In order to be usable in the RepeatExplorer2 (RE2) analysis, the Illumina reads must meet certain requirements, such as having a certain length and being provided in a certain file format. (B) The RE2 results (graph-based clusters) of the analysis using *H. visseri* read data were used for the generation of a custom database containing the most abundant repeats within the *H. visseri* genome.

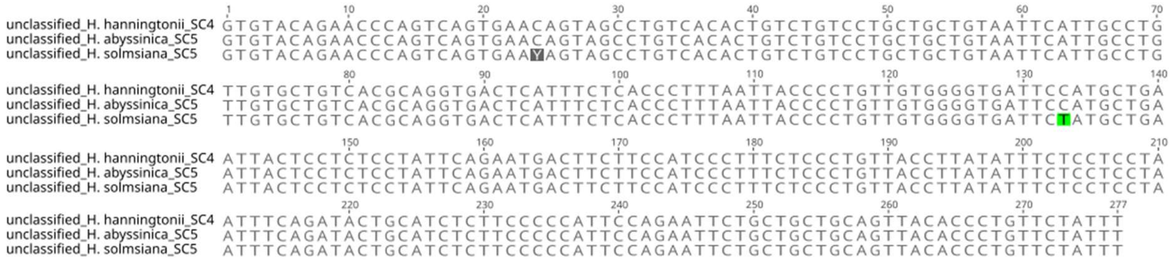

Fig. S2 Alignment of the consensuses of the highly abundant unclassified read clusters from the *H. abyssinica*, *H. hanningtonii*, and *H. solmsiana* individual analysis results. The contigs from the specific RE2 cluster were aligned to the highest read depth contig from the cluster to refine the consensuses. The repeat consensuses have the same length of 277 bp, and one nucleotide substitution and one potential nucleotide substitution were observed from the consensus alignment. The repeat consensuses could neither be identified using the RE2 database, nor by using publicly available databases.

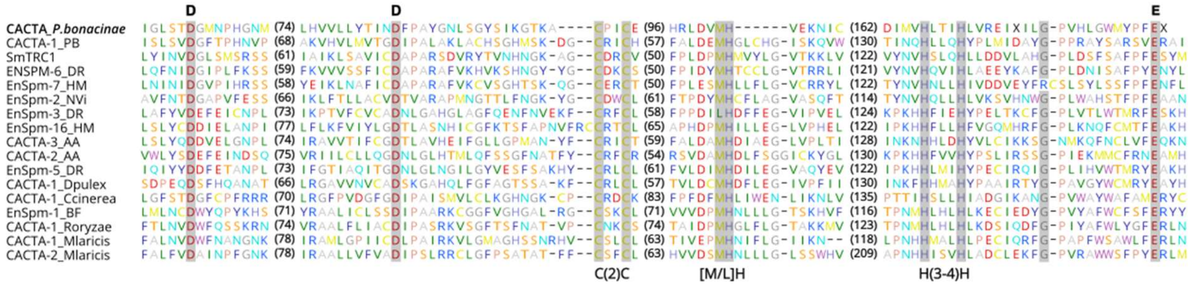

Fig. S3 Alignment of the transposase amino acid sequences of the highly abundant En/Spm_CACTA DNA transposon from the *P. bonacinae* genome and 16 further En/Spm_CACTA DNA transposon sequences, which represent this transposon superfamily, from Yuan & Wessler (2011). The pivotal catalytic region represented by three amino acids (D, D, E) and the additional conserved motifs: C(2)C, [M/L]H, and H(3-4)H are shaded.

Aristolochia contorta
Minimum Free Energy = -42.3
GC content: 54.5%

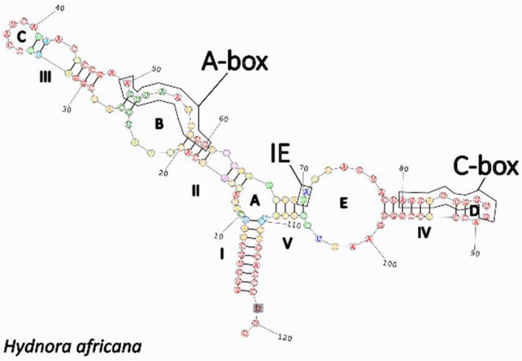

Hydnora visseri / Hydnora longicollis
Minimum Free Energy = -41.4
GC content: 52.9%

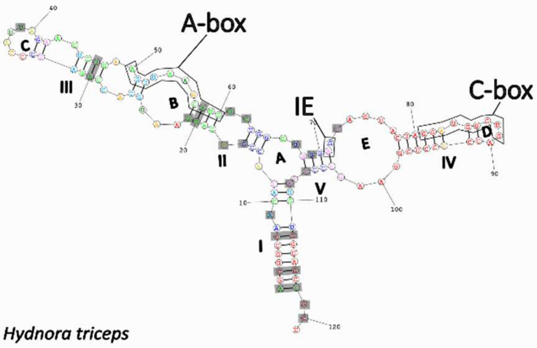

Hydnora africana
Minimum Free Energy = -41.4
GC content: 53.7%

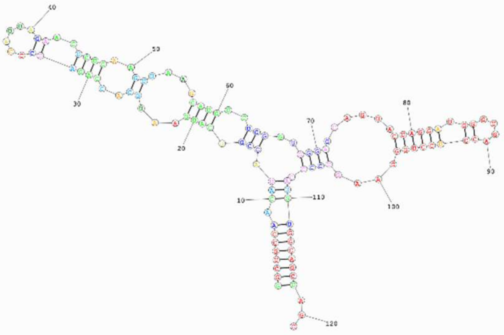

Hydnora triceps
Minimum Free Energy = -38.6
GC content: 52.1%

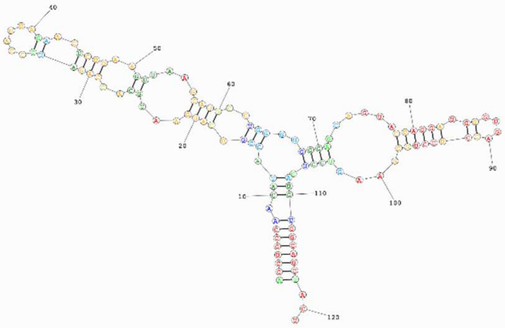

Hydnora abyssinica
Minimum Free Energy = -41.4
GC content: 54.2%

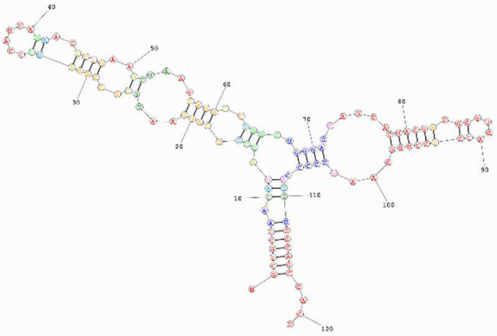

Hydnora solmsiana / Hydnora hanningtonii
Minimum Free Energy = -43.3
GC content: 53.7%

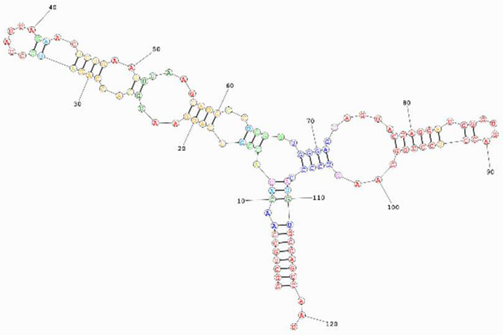

Hydnora esculenta
Minimum Free Energy = -41.4
GC content: 52.9%

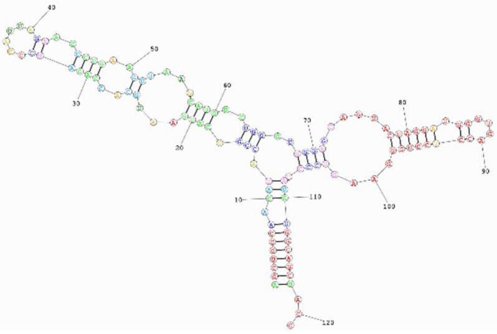

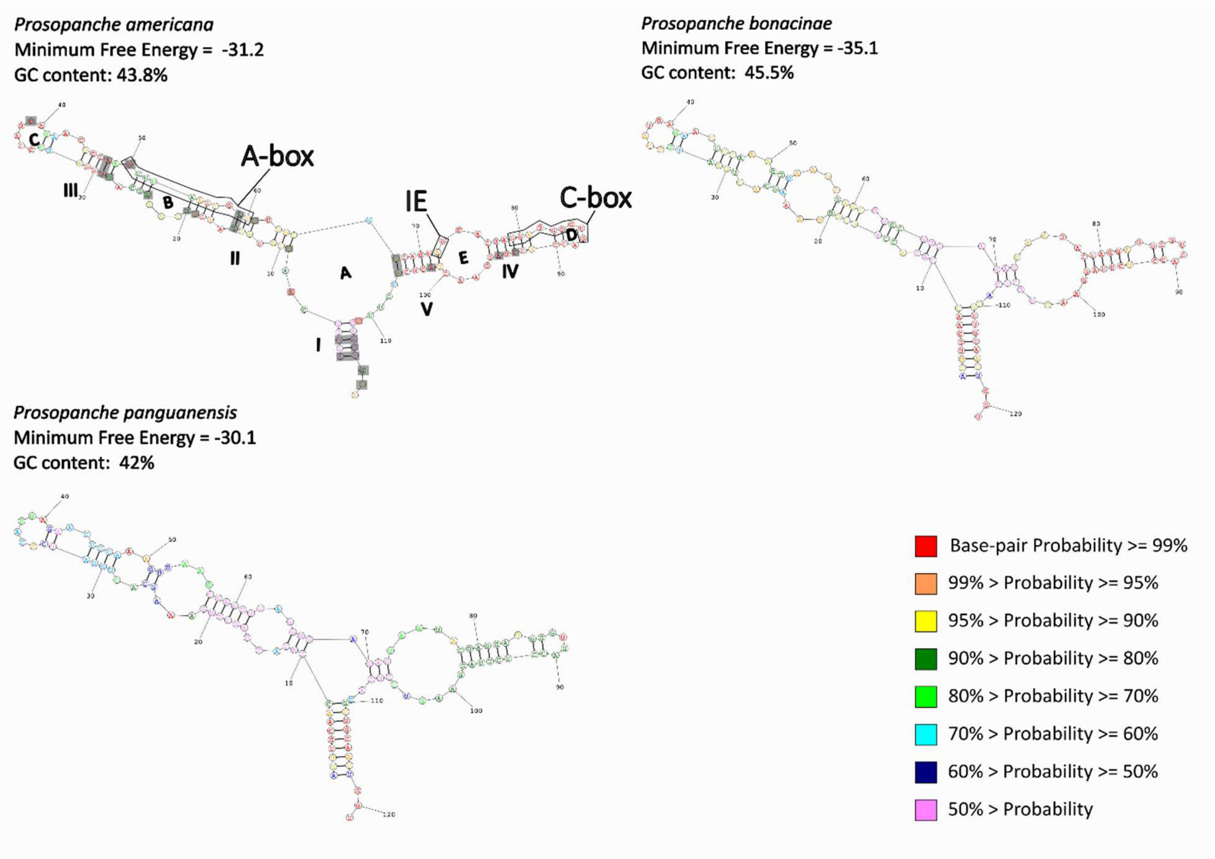

Fig. S4 Secondary structure of Hydnoraceae 5S rRNAs. The structures were reconstructed using the RNAstructure software (Reuter & Mathews, 2010). The functional structures (helices I – IV and loops A – E) were described based on the general secondary structure model of eukaryotic 5S rRNAs (Barciszewska *et al.*, 2000) and the secondary structure of the major 5S rRNA from *A. thaliana* (Cloix *et al.*, 2003). 5S rRNA secondary structure of *Aristolochia concorta* was included for a comparison. Gray shadings indicate (complementary) substitutions, corresponding to point mutations highlighted in the 5S rDNA alignment (Fig. S6).

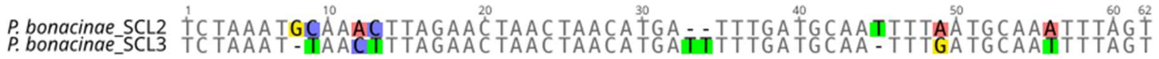

Fig. S5 Alignment of the RE2-provided consensuses from two highly abundant read clusters representing *P. bonacinae*-specific satellite DNAs. In the *P. bonacinae* individual analysis, two highly abundant read clusters (supercluster 2 and 3; SCL2/3) were identified as satellite DNAs. The alignment revealed the same length (60 bp) of both consensuses, potentially originating from a single ancestral sequence, diverged due to nucleotide substitutions as well as potential insertion/deletion mutations.

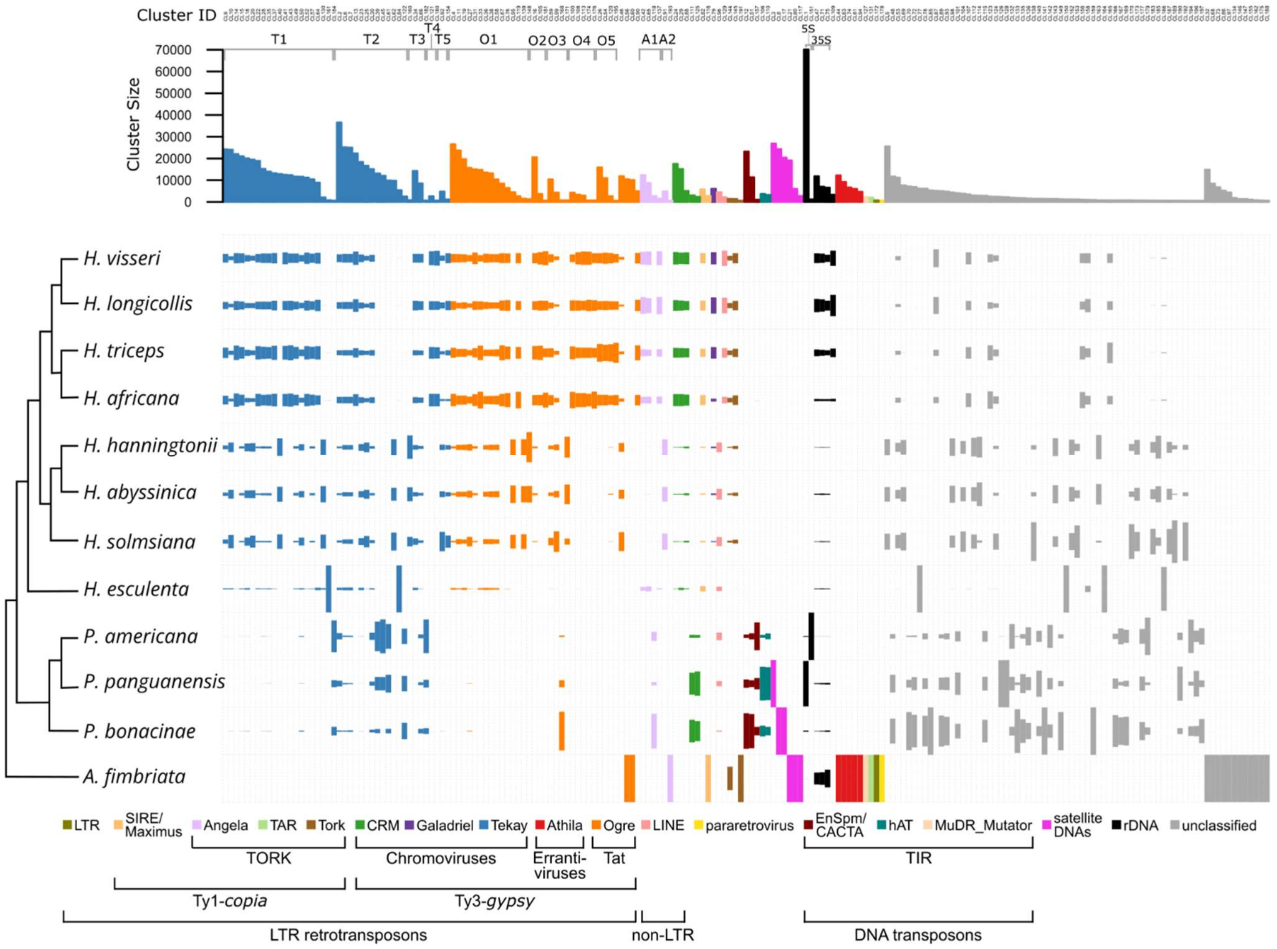

Fig. S6 Comparative genomic repeat composition among eleven Hydnoraceae species and *A. fimbriata*. The size of each rectangle is proportional to the genomic abundance of the repeat in the respective species. Dashed vertical lines group read clusters that belong to the same repeat, while black arrows indicate taxa-specific sequence variants of *Hydnora*Tekay1. In the top bar chart, the height of

each bar represents the number of reads in each cluster comprising at least 516 reads (≥ 0.01% of the analyzed reads). Clusters corresponding to the same repetitive element are sorted by read count, from highest to lowest, so the bar chart depicts the ranked abundance of each repetitive element. The cladogram is adapted from Mkala *et al.* (2023), modified. The figure is modified from Fig. 5 to include cluster identifiers.

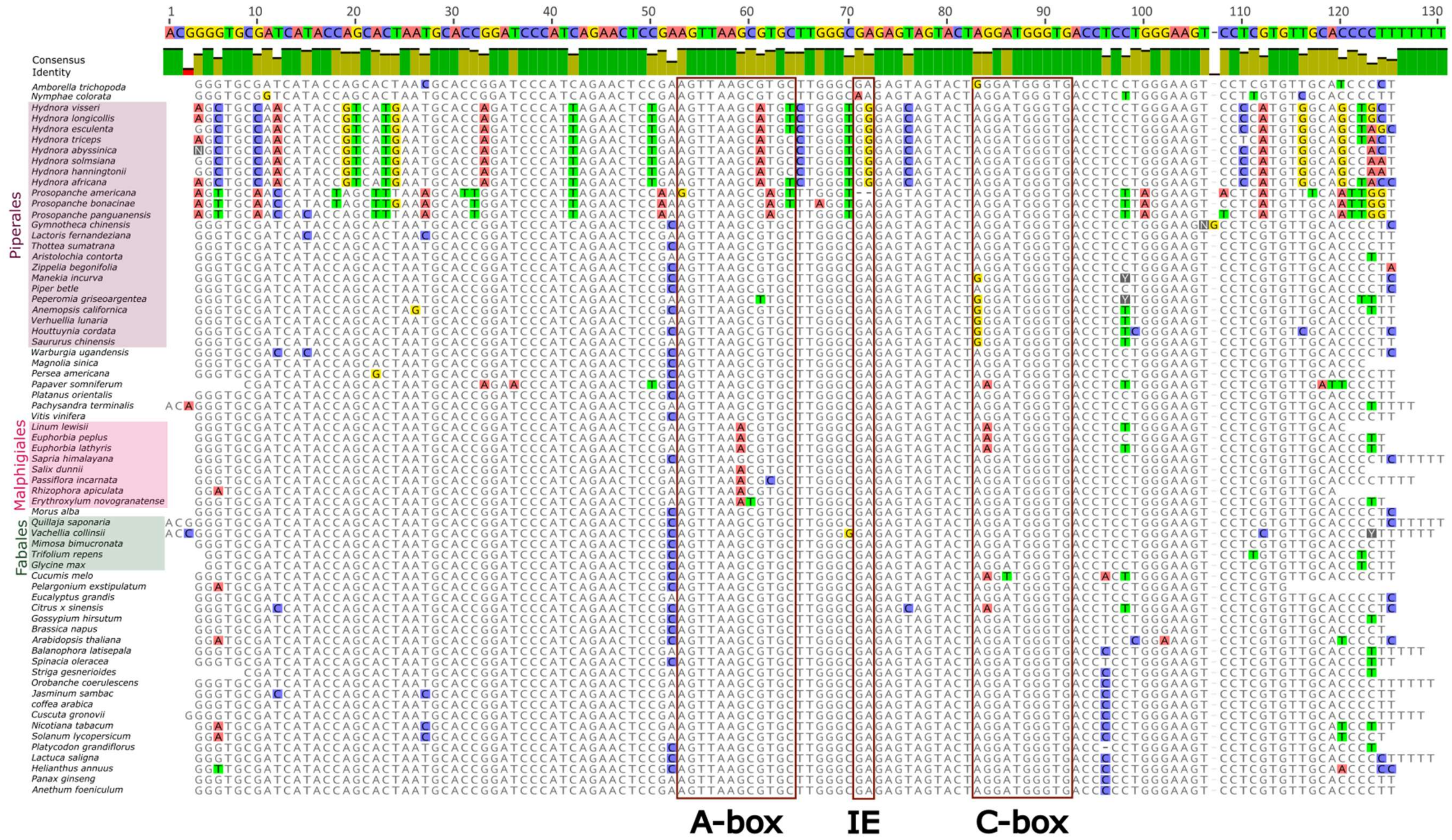

Fig. S7 Alignment of 5S rDNAs of Hydnoraceae and of 56 further angiosperms. Disagreements from the overall consensus sequence (given on top) are highlighted (A = red, T = green, C = blue, G = yellow). The highly conserved internal control region (ICR) for transcription (A-box, Intermediate element=IE, C-box) is indicated in red boxes referring to (Cloix *et al.*, 2003).

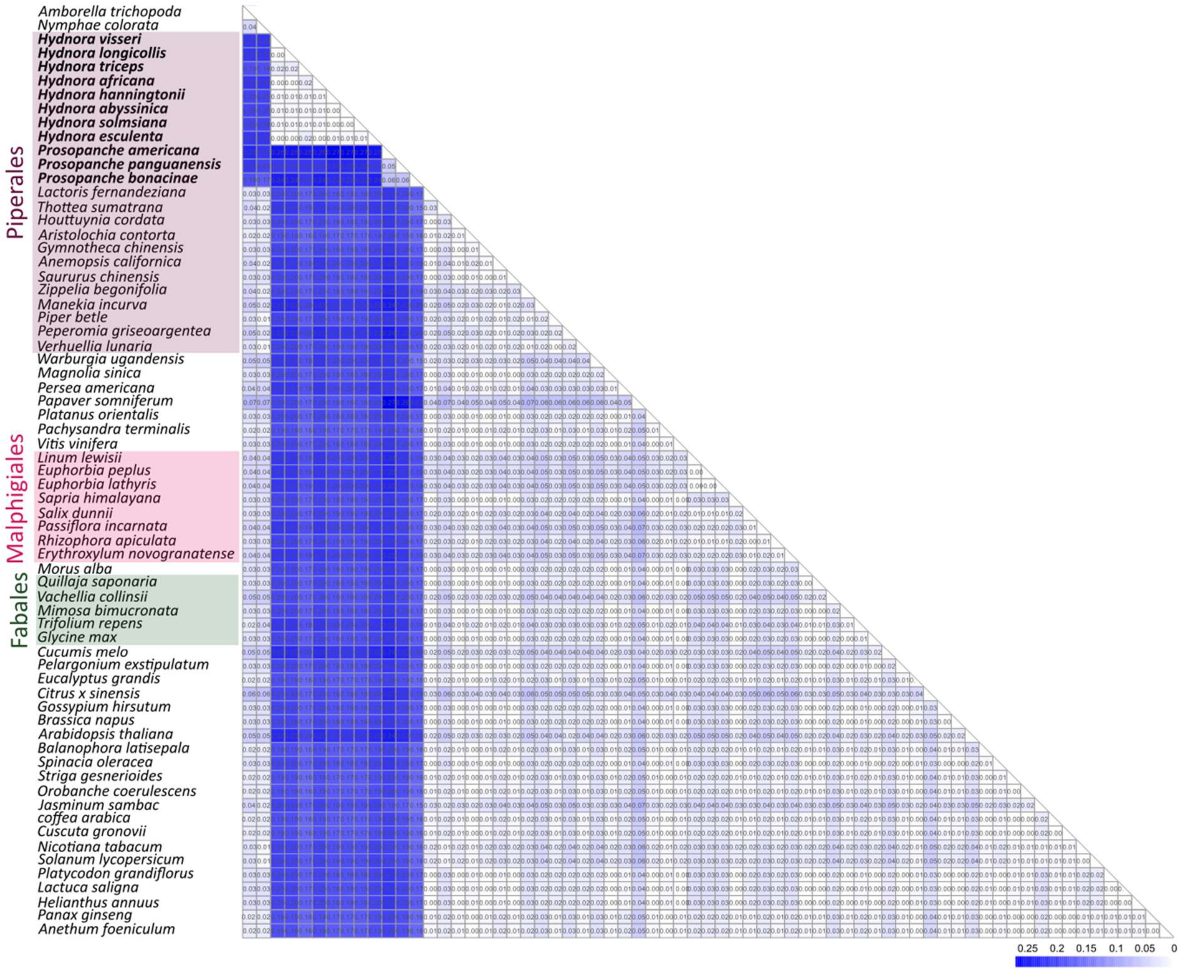

Fig. S8 Genetic distances between the coding regions of Hydnoraceae 5S ribosomal DNAs and those of 56 other angiosperms, calculated using the Kimura-2-parameter model (Kimura, 1980). The resulting distance matrix ranges from 0 to 0.27, with higher values indicating greater sequence variation. Representatives of the order Piperales are highlighted in purple. Fabales and Malpighiales, the host orders, are highlighted in green and pink, respectively. Parasitic plants are highlighted in bold. The figures is modified from Fig. 7 to contain the values of genetic distances.

Table S1 Plant material, DNA extraction and genome sequencing

Taxon	Taxon Codes	DNA extraction method	Library preparation	Sequencing platform	Read length	Reference	NCBI identifiers
Hydnora visseri	Hvis	DNeasy Plant Mini Kit (Qiagen)	NEBNext Ultra DNA	Illumina HiSeq-2000	100	Naumann *et al.* (2016)	SRR31419648
		Modified (Doyle & Doyle, 1987) adding RNAse A (Thermo Scientific, Waltham, MA, USA) treatment (10 mg/ml)	BluePippin™ Size-Selection System	43 PacBio RS2 SMRT cells v3	500 – 71,657	This study	-
Hydnora africana	Hafr	Modified (Doyle & Doyle, 1987) adding RNAse A (Thermo Scientific, Waltham, MA, USA) treatment (10 mg/ml)	NEBNext Ultra DNA	Illumina HiSeq 2500 RapidMode	150	Jost *et al.* (2022)	SRR31419652
Hydnora longicollis	Hlon	DNeasy Plant Maxi Kit (Qiagen)	NEBNext Ultra DNA	Illumina HiSeq 2500 RapidMode	150	Jost *et al.* (2022)	SRR31419650
Hydnora triceps	Htri	Modified (Doyle & Doyle, 1987) adding RNAse A (Thermo Scientific, Waltham, MA, USA) treatment (10 mg/ml)	NEBNext Ultra DNA	Illumina HiSeq 2500 RapidMode	150	Jost *et al.* (2022)	SRR31419649
Hydnora abyssinica	Haby		Illumina TruSeq DNA	Illumina HiSeq-2000	150	Mkala *et al.* (2023)	SRR31398320
Hydnora hanningtonii	Hhan		NEBNext Ultra DNA	Illumina HiSeq 2500 RapidMode	150	Jost *et al.* (2022)	SRR33089576
Hydnora solmsiana	Hsol		NEBNext Ultra DNA	Illumina HiSeq 2500 RapidMode	150	Jost *et al.* (2022)	SRR33089575
Hydnora esculenta	Hesc		NEBNext Ultra DNA	Illumina HiSeq 2500 RapidMode	150	Jost *et al.* (2022)	SRR31419651
Prosopanche americana	Pame	DNeasy Plant Maxi Kit (Qiagen, Venlo, Netherlands)	Illumina TruSeq DNA	Illumina NextSeq High Output	150	Jost *et al.* (2020)	SRR31419647
Prosopanche bonacinae	Pbon	Modified (Doyle & Doyle, 1987) adding RNAse A (Thermo Scientific, Waltham, MA, USA) treatment (10 mg/ml)	NEBNext Ultra DNA	Illumina HiSeq RapidMode	150	Jost *et al.* (2022)	SRR31419646
Prosopanche panguanensis	Ppan		NEBNext Ultra DNA	Illumina HiSeq RapidMode	150	Jost *et al.* (2022)	SRR31419645

Table S2 Reconstruction of repetitive elements within the *H. visseri* genome

SCL*	Pipeline (RE2) provided Annotation	Annotated protein domain	Manual Annotation	SCL *	Pipeline (RE2) provided Annotation	Annotated protein domain	Manual Annotation
1	Tekay	*gag-pol* polyprotein**	*Hydnora*Tekay1	17	Ogre	INT	*Hydnora*Ogre3
2	Tekay	*gag-pol* polyprotein**	*Hydnora*Tekay2	18	all	-	Ogre (unassigned)
3	Ogre	*gag-pol* polyprotein***	*Hydnora*Ogre1	19	all	GAG	*Hydnora*Ogre5
4	all	-	*Hydnora*Ogre1	20	all	-	*Hydnora*Ogre2
5	CRM	RT	*Hydnora*CRM1	21	satellite DNA	INT	*Hydnora*Galadriel1
6	35S rDNA	-	35S rDNA	22	SIRE	RT	*Hydnora*SIRE1
7	Angela	RH	*Hydnora*Angela1	23	Ogre	GAG	*Hydnora*Ogre2
8	CRM	INT	*Hydnora*CRM2	24	Ogre	INT, RT	*Hydnora*Ogre4
9	Tekay	RT	*Hydnora*Tekay3	25	all	-	all
10	all	-	*Hydnora*Ogre2	26	all	GAG	*Hydnora*Tekay4
11	all	-	all	27	Ogre	INT	*Hydnora*Ogre5
12	LTR retro-transposon	-	*Hydnora*Ogre2	28	all	-	*Hydnora*Ogre3
13	all	-	*Hydnora*Ogre1	29	Tekay	INT	*Hydnora*Tekay5
14	all	-	*Hydnora*Ogre5	30	all	-	*Hydnora*Ogre2
15	Tat	RT	*Hydnora*Ogre2	31	Ogre	GAG	*Hydnora*Ogre4
16	all	-	all	32	Angela	RT	*Hydnora*Angela2

*From the RE2 individual analysis results of *H. visseri*, superclusters (SCL) comprising more than 10,000 reads (0.2% of the analyzed reads)were taken into account for the reconstruction of reference sequences.

**Showing similarity to the chromodomain (CHD), the group-specific antigen (GAG), the integrase (INT), the protease (PROT), the ribonuclease H1 (RH), and the reverse transcriptase (RT). For the principals of annotations embedded in RE2, see (Novák *et al.*, 2013, 2020).

***Showing similarities to GAG, INT, PROT, RH, RT, and the archeal ribonuclease H1 (aRH)

Table S3 Hydnoraceae short read mapping to 5S rDNA references*

Species	Number of Illumina reads	Number of mapped reads	[%]
H. visseri	95,483,059	291	0.00030
H. longicollis	257,184,941	122	0.00005
H. triceps	8,543,515	231	0.00270
H. africana	13,858,819	87	0.00063
H. hanningtonii	16,130,357	25	0.00016
H. abyssinica	23,408,887	45	0.00019
H. solmsiana	13,096,372	152	0.00116
H. esculenta	18,977,608	39	0.00021
*P. americana***	186,620,441	265,644	0.14234
Aristolochia fimbriata	60,754,989	45,193	0.07439

*Proportion of reads from each *Hydnora* short read dataset that mapped to the 5S rDNA reference from *H. triceps* using Bowtie2. The reads are concordantly or disconcordantly aligned onto the 5S rDNA reference sequence, using mixed mode (default setting in Bowtie2). The number of aligned reads were viewed and counted using Geneious v.6.1.8 (Kearse *et al.*, 2012). For comparison, short reads of a close photoautotrophic relative of the Hydnoraceae, *Aristolochia fimbriata* (Aristolochiaceae, SRR13748080) were mapped to the 5S rDNA reference from *H. triceps* as well.

***P. americana* short reads were mapped to the 5S rDNA reference from *P. panguanensis* using the same Bowtie2 settings.

Table S4 NCBI identifiers for the genome sequencing data for the reconstructed angiosperms 5S rDNAs (separate Excel file is provided)

Table S5 Relative rate test among 5S rDNA of *H. visseri* and closely related species

Outgroup	Ingroup 1	Ingroup 2	Identical sites	Divergent sites in all three sequences	Unique differences to Ingroup 1	Unique differences to Ingroup 2	Unique differences outgroup	χ2 test statistic*	p-value
Arabidopsis thaliana	*Hydnora visseri*	*Aristolochia contorta*	93	3	21	0	4	21	<0.00001
Arabidopsis thaliana	*Hydnora visseri*	*Lactoris fernandeziana*	92	2	24	0	2	24	<0.00001
Arabidopsis thaliana	*Hydnora visseri*	*Thottea sumatrana*	91	2	22	2	4	16.67	0.00004
Arabidopsis thaliana	*Hydnora visseri*	*Propanche americana*	79	5	10	13	12	0.39	0.53161

*The chi-square test is based on 1 degree of freedom

Table S6 Summary of genomic proportions of repetitive elements in *Hydnora* genomes

Repeat proportion (%)		*H. visseri*	*H. longicollis*	*H. triceps*	*H. africana*	*H. hanningtonii*	*H. abyssinica*	*H. solmsiana*	*H. esculenta*	Mean
Class I	Ty3-*gypsy*	37.4	31.6	21.6	40.2	22.5	22.3	28.5	9.7	26.7
	Ty1-*copia*	2.4	2.5	0.7	1.9	1.5	1.4	1.9	1.3	1.70
	LINEs	0.25	0.23	0.19	0.07	0.00	0.25	0.25	0.00	0.22
	Pararetrovirus	0.14	0.02	0.07	0.14	0.00	0.00	0.00	0.00	0.08
Class II	DNA transposons	0.00	0.00	0.00	0.00	0.10	0.10	0.23	0.47	0.23
Tandem repeats	35S rDNA	1.41	1.98	0.54	0.38	0.07	0.14	0.12	0.07	0.59
	5S rDNA	0.00	0.00	0.01	0.00	0.00	0.00	0.00	0.00	0.00
	satellite DNAs	0.00	0.00	0.05	0.00	0.47	0.00	0.02	0.07	0.13
Unclassified repeats		14.69	20.38	26.63	10.7	14.66	21.9	18.77	23.38	18.89
Total repeat proportion		56.25	56.73	49.77	52.99	39.34	46.06	49.78	34.55	48.18

Table S7 Summary of genomic proportions of repetitive elements in *Prosopanche* genomes

Repeat proportion (%)		*P. americana*	*P. panguanensis*	*P. bonacinae*	Mean
Class I	Ty3-*gypsy*	16.66	7.63	9.38	11.22
	Ty1-*copia*	1.11	0.00	0.84	0.98
	Pararetrovirus	0.00	0.00	0.00	0.00
	LINEs	0.33	0.05	0.06	0.15
Class II	DNA transposons	2.62	1.84	7.96	4.14
	Helitron	0.03	0.00	0.00	0.03
	35S rDNA	0.00	0.09	0.12	0.11
	5S rDNA	0.55	15.71	0.41	5.56
	satellite DNAs	0.2	6.89	10.88	5.99
	Unclassified repeats	21.57	14.31	22.82	19.57
Total repeat proportion		43.07	46.52	52.48	47.36

Table S8 Relative genomic abundance of specific Hydnoraceae repeats

Relative abundance of shared repeats* [%]	T1	T2	T3	T4	T5	O1	O2	O3	O4	O5
H. visseri	17.0	13.3	18.1	30.0	13.3	17.0	22.6	12.9	22.8	22.2
H. longicollis	14.9	11.4	19.3	18.5	14.1	14.7	18.8	9.1	21.4	18.9
H. triceps	21.3	9.0	10.2	24.8	7.6	18.3	28.6	14.3	24.2	36.4
H. africana	18.6	10.7	11.7	26.6	4.1	21.6	21.8	16.0	31.5	22.2
H. hanningtonii	8.7	8.0	8.2	0.0	11.2	8.9	3.3	10.2	0.0	0.1
H. abyssinica	8.1	7.2	7.6	0.0	11.5	8.9	3.3	10.4	0.0	0.2
H. solmsiana	9.3	8.4	10.3	0.1	38.0	8.0	1.5	22.5	0.0	0.1
H. esculenta	1.5	5.4	3.1	0.0	0.0	2.3	0.0	0.2	0.0	0.0
P. americana	0.3	14.9	4.8	0.0	0.0	0.0	0.0	0.1	0.0	0.0
P. panguanensis	0.1	8.8	2.8	0.0	0.0	0.1	0.0	0.7	0.0	0.0
P. bonacinae	0.3	3.0	3.9	0.0	0.3	0.1	0.0	3.7	0.0	0.0
A. fimbriata	0.0	0.0	0.0	0.0	0.0	0.0	0.0	0.0	0.0	0.0
Num. of reads	286663	212563	23375	3200	5890	176928	24801	16044	11204	29876

Relative abundance of shared repeats* [%]	En/Spm _CACTA	hAT	satel-lite DNAs	5S	35S					
H. visseri	0.0	0.0	0.0	0.0	20.4					
H. longicollis	0.0	0.0	0.0	0.0	27.9					
H. triceps	0.0	0.0	0.0	0.0	13.4					
H. africana	0.0	0.0	0.0	0.0	4.3					
H. hanningtonii	0.0	0.0	0.0	0.0	0.8					
H. abyssinica	0.0	0.0	0.0	0.0	1.9					
H. solmsiana	0.0	0.0	0.0	0.0	1.3					
H. esculenta	0.0	0.0	0.0	0.0	0.9					
P. americana	10.5	9.0	0.0	3.5	0.2					
P. panguanensis	16.9	70.5	27.1	94.1	2.0					
P. bonacinae	72.6	20.4	45.0	2.4	1.5					
A. fimbriata	0.0	0.0	28.0	0.1	25.4					
Num. of reads	35514	6840	99229	71275	28658					

*During the RE2 comparative analysis, the pre-labelled reads from all Hydnoraceae species are jointly clustered and annotated according to the reference repeat database (reconstructed from the *H. visseri* genome). Subsequently, the pooled reads per each repeat (‘Num. of reads’ in the table) were again classified according to the species-specific code, aiming to reveal the relative genomic abundance of shared repeats and other abundant repeats in Hydnoraceae genomes.

References

Cloix C, Yukawa Y, Tutois S, Sugiura M, Tourmente S. **2003**. In vitro analysis of the sequences required for transcription of the *Arabidopsis thaliana* 5S rRNA genes. *The Plant Journal* **35**: 251–261.

Doyle JJ, Doyle JL (Eds.). **1987**. A rapid DNA isolation procedure for small quantities of fresh leaf tissue. *Phytochemical Bulletin*.

Hatt SA, Cameron DD, Grace OM, Rocamundi N, Cocucci AA, Martel C, Thorogood CJ. **2023**. *Prosopanche*: A remarkable genus of parasitic plants. *PLANTS, PEOPLE, PLANET* **5**: 163–168.

Hatt SA, Thorogood CJ, Bolin JF, Musselman LJ, Cameron DD, Grace OM. **2022**. A taxonomic revision of the genus *Hydnora* (Hydnoraceae). : 2022.10.13.512068.

Jost M, Naumann J, Bolin JF, Martel C, Rocamundi N, Cocucci AA, Lupton D, Neinhuis C, Wanke S. **2022**. Structural plastome evolution in holoparasitic Hydnoraceae with special focus on inverted and direct repeats. *Genome Biology and Evolution* **14**: evac077.

Jost M, Naumann J, Rocamundi N, Cocucci AA, Wanke S. **2020**. The first plastid genome of the holoparasitic genus *Prosopanche* (Hydnoraceae). *Plants* **9**: 306.

Mkala EM, Jost M, Dong X, Mwachala G, Musili PM, Wanke S, Hu G-W, Wang Q-F. **2023**. Phylogenetic and comparative analyses of *Hydnora abyssinica* plastomes provide evidence for hidden diversity within Hydnoraceae. *BMC Ecology and Evolution* **23**: 34.

Mkala EM, Mutungi MM, Mutinda ES, Oulo MA, Wanga VO, Mwachala G, Hu G-W. **2021**. Understanding the ethnobotany, chemistry, pharmacology, and distribution of genus *Hydnora* (Aristolochiaceae). *Plants* **10**: 494.

Naumann J, Der JP, Wafula EK, Jones SS, Wagner ST, Honaas LA, Ralph PE, Bolin JF, Maass E, Neinhuis C, *et al.* 2016. Detecting and characterizing the highly divergent plastid genome of the nonphotosynthetic parasitic plant *Hydnora visseri* (Hydnoraceae). *Genome Biology and Evolution* **8**: 345–363.

Novák P, Neumann P, Macas J. **2020**. Global analysis of repetitive DNA from unassembled sequence reads using RepeatExplorer2. *Nature Protocols* **15**: 3745–3776.

Novák P, Neumann P, Pech J, Steinhaisl J, Macas J. **2013**. RepeatExplorer: a Galaxy-based web server for genome-wide characterization of eukaryotic repetitive elements from next-generation sequence reads. *Bioinformatics* **29**: 792–793.

Reuter JS, Mathews DH. **2010**. RNAstructure: software for RNA secondary structure prediction and analysis. *BMC Bioinformatics* **11**: 129.

Yuan Y-W, Wessler SR. **2011**. The catalytic domain of all eukaryotic cut-and-paste transposase superfamilies. *Proceedings of the National Academy of Sciences* **108**: 7884–7889.
